# Chimeric *SFT2D2‐TBX19* Promotes Prostate Cancer Progression by Encoding TBX19‐202 Protein and Stabilizing Mitochondrial ATP Synthase through ATP5F1A Phosphorylation

**DOI:** 10.1002/advs.202408426

**Published:** 2024-11-14

**Authors:** Chenxi Hu, Zaosong Zheng, Shiyu Pang, Yuanchao Zhu, Jirong Jie, Zhuocheng Lai, Xiangbo Zeng, Yongyuan Xiao, Zhifeng Chen, Jingjing Zhao, Yuejun Du, Fei Li, Qiong Wang, Wanlong Tan

**Affiliations:** ^1^ Department of Urology Nanfang Hospital Southern Medical University Guangzhou Guangdong 510515 P. R. China; ^2^ School of Traditional Chinese Medicine Southern Medical University Guangzhou Guangdong 510515 P. R. China; ^3^ Southern Medical University Guangzhou Guangdong 510515 P. R. China; ^4^ Department of Urology Sun Yat‐Sen Memorial Hospital Sun Yat‐Sen University Guangzhou 510120 P. R. China

**Keywords:** ATP synthase, chimeric RNA, mitochondrial stability, prostate cancer, *SFT2D2‐TBX19*

## Abstract

Specific chimeric RNAs and their products are consistently regarded as ideal tumor diagnostic markers and therapeutic targets. Chimeric RNAs can mediate tumor cell plasticity, neuroendocrine processes, polarization of tumor‐associated macrophages, and resistance to chemotherapy and immunotherapy. However, the discovery of chimeric RNAs in prostate cancer is still in its early stages. This study identifies the chimeric *SFT2D2‐TBX19* as a novel transcript encoding the TBX19‐202 protein. Both TBX19‐202 and its parental TBX19, which share homologous amino acid sequences, enhance prostate cancer cell proliferation, migration, and invasion. Additionally, *SFT2D2‐TBX19* also functions as a lncRNA, interacting with the ATP synthase F1 subunit ATP5F1A, thereby increasing ATP5F1A phosphorylation mediated by TNK2/ACK1, which stabilizes the interaction between ATP5F1A and ATP5F1B. The region spanning 1801‐2400 bp of *SFT2D2‐TBX19* and the intermediate structural domain of ATP5F1A are crucial functional areas. This stabilization of ATP5F1A and ATP5F1B enhances mitochondrial ATP synthase activity and ATP production. Even under conditions of mitochondrial vulnerability, *SFT2D2‐TBX19* protects mitochondrial structural stability to maintain prostate cancer cell proliferation. This research provides comprehensive evidence that chimeric *SFT2D2‐TBX19* promotes prostate cancer progression by encoding the TBX19‐202 protein and stabilizing mitochondrial ATP synthase via ATP5F1A phosphorylation. These findings highlight *SFT2D2‐TBX19* as a potential therapeutic target for prostate cancer.

## Graphical abstract

*SFT2D2‐TBX19* functions as a lncRNA capable of interacting with the ATP synthase F1 subunit ATP5F1A, enhancing its phosphorylation via TNK2/ACK1, which in turn stabilizes the interaction between ATP5F1A and ATP5F1B. Additionally, *SFT2D2‐TBX19* could also encode TBX19‐202, which shares a similar oncogenic role with the parental TBX19 in promoting the progression of prostate cancer.

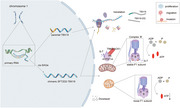

## 1 Introduction

Specific gene fusions and their generated fusion products, including chimeric RNAs and proteins, have long been recognized as ideal diagnostic markers and therapeutic targets for tumors. The well‐known *BCR‐ABL* fusion, in particular, has become a crucial reference standard for the diagnosis and treatment of chronic myeloid leukemia (CML).^[^ ^1^ ^]^ The chromosomal rearrangement involving the *TMPRSS2‐ERG* gene fusion, which is universally present in prostate cancer, shows potential for detecting aggressive forms of the disease.^[^ ^2^ ^]^ Additionally, there are *EML4‐ALK* in lung adenocarcinomas,^[^ ^3^ ^]^ *FGFR3‐TACC3* in glioblastoma multiforme (GBM),^[^ ^4^ ^]^ among others.

Chimeric RNAs are hybrid, discontinuous transcripts that include exons from two separate parental genes. Chimeric RNAs can be produced through three formation mechanisms: chromosomal rearrangement, trans‐splicing and cis‐splicing.^[^ ^5^, ^6^, ^7^ ^]^ In terms of noncoding functions, they can act as long noncoding RNAs or circle RNAs to mediate cellular metabolism.^[^ ^8^ ^]^ Regarding coding functions, they can encode fusion or novel proteins or regulate the expression of parental genes through their translational regulation regions.^[^ ^5^ ^]^ Our previous studies also revealed that e2e4 *TMPRSS2‐ERG* variant exhibits enzalutamide resistance, a characteristic that distinguishes it from the conventional e1e4 chimeric form.^[^ ^9^ ^]^ Recent studies suggest that chimeric RNAs often emerge in the later stages of cancers, particularly after drug treatments. Their emergence may signify the adaptive evolution of drug‐resistant cancer cells. For instance, specific chimeric RNAs like *MYH9‐EIF3D* and *LDLR‐RPL31P11* have been identified in docetaxel‐resistant prostate cancer, while *KIF5B‐RET* has been observed in non‐small cell lung carcinoma resistant to EGFR tyrosine kinase inhibitors. These suggest that chimeric RNAs may contribute to generating new functionality that facilitates cancer cells to survive.^[^ ^10^, ^11^ ^]^ However, research on chimeric RNAs of prostate cancer is still in its early stages. Our aim is to discover more specific chimeras, which provides references for exploring new targets for the diagnosis and treatment of prostate cancer.

## 2 Results

### 2.1 The Discovery of a Novel Chimeric *SFT2D2‐TBX19* in Prostate Cancer

**Figure 1:**
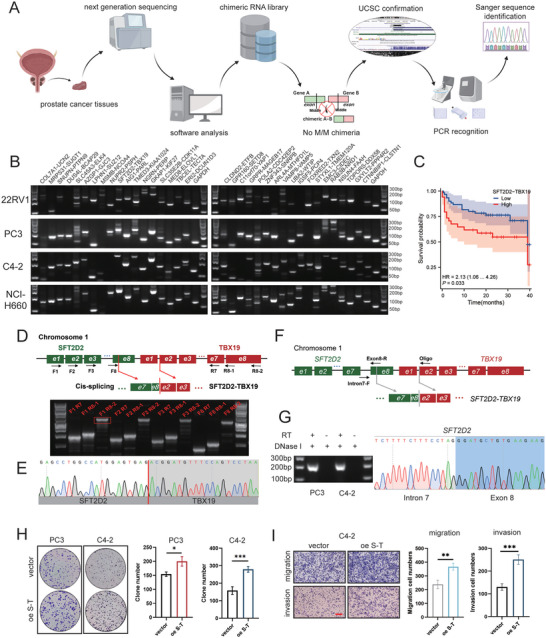
The discovery of a novel oncogenic chimeric *SFT2D2‐TBX19* in prostate cancer. A) The pipeline for discovering chimeric RNAs. B) Validation of 58 chimeric RNAs in NCI‐H660, 22RV1, PC3 and C4‐2 cell lines (36 chimeras are listed here). C) Survival analysis comparing high and low expression groups of *SFT2D2‐TBX19* in the CPGEA database. D) Primer design, PCR identification, and analysis of *SFT2D2‐TBX19* full length. Green and red blocks represent exons, black lines represent introns or intergenic regions. The arrowheads indicate the forward and reverse primers used for PCR amplification. E) Sanger sequencing validated *SFT2D2‐TBX19* junction sequence obtained from PCR full length products. F) The schematic diagram for validating cis‐splicing between adjacent genes (cis‐SAGe). Green and red blocks represent exons, while black lines represent introns or intergenic regions. The arrowhead indicates the oligo primer used for reverse transcription. Intron 7‐F and exon 8‐R primers were used to amplify the junction seq between intron 7 and exon 8 of *SFT2D2*. G) (left panel) RNAs from C4‐2 and PC3 were first treated with DNaseI. The correct product was only seen in RNA samples with Reverse Transcriptase (RT); (right panel) Sanger sequencing validated the PCR products. H) Colony formation of PC3, C4‐2 cells following the overexpression of *SFT2D2‐TBX19*. I) Migration and invasion assays of C4‐2 cells following the overexpression of *SFT2D2‐TBX19*. The scale bar in the lower left corner represents 200 µm. C: Cox proportional‐hazards model analysis; H,I: Data are represented as mean ± SD (*n* = 3 replicates). Student's t test was used to determine statistical significance, *p < 0.05, **p < 0.01, ***p < 0.001.

We filtered chimeric RNAs from the CPGEA database (http://www.cpgea.com) to construct a chimeric RNA library. We have completed the screening and presented the list of chimeric RNAs at the CUA2022 conference.^[^ ^12^ ^]^ Here, we selected 58 chimeric RNAs from that list, which were validated in RNA transcript mixtures from prostate cancer cell lines for further screening. Subsequently, 58 chimeric RNAs were validated in six available prostate cancer cell lines and prostatic epithelial cell line RWPE‐1 through RT‐qPCR amplification of the specific junction sequences for each chimeric RNA (**Figures** **1**A,B and S1A,B, Supporting Information). And their genetic characteristics were detailed in Table S1 (Supporting Information). Notably, we identified that 16 of the 58 chimeric RNAs were specifically expressed, while 32 were highly expressed in prostate cancer cell lines. Among the 48 chimeric RNAs mentioned, AGREP analysis (https://www.tgries.de/agrep/) was performed to quantify their expression by searching for specific junction sequences in the CPGEA RNA‐seq data, as described in our previous study.^[^ ^8^ ^]^ Only *SFT2D2‐TBX19* and *ERV3‐1‐ZNF506* were associated with poor prognosis of prostate cancer patients (Figure 1C and Figure S1C, Supporting Information). Here we focused on chimeric *SFT2D2‐TBX19*, which was validated as highly expressed in 21 paired tumor samples that we collected previously from prostate cancer patients^[^ ^13^ ^]^ (Figure S1D, Supporting Information). Interestingly, the transcription level of *SFT2D2‐TBX19* was highest in the neuroendocrine prostate cancer (NEPC) cell line NCI‐H660 (Figure S1E, Supporting Information), suggesting that *SFT2D2‐TBX19* may be correlated with the malignant progression of prostate cancer.

### 2.2 *SFT2D2‐TBX19* Is Formed by the First 8 Exons of the Parental *SFT2D2* and the Last 7 Exons of the Parental *TBX19*, and Is Presumed to Be a Product of Cis‐SAGe

Since *SFT2D2‐TBX19* is a novel chimeric RNA that has not been previously reported, we employed a permutation and combination approach for PCR to determine its full length (Figure 1D). Even with the combination of a forward primer closest to the 5′ end of *SFT2D2* and a reverse primer closest to the 3′ end of *TBX19* (F1, R8‐2), we successfully amplified the full length of *SFT2D2‐TBX19*. After recovering the amplification products, we identified the junction sequence using Sanger sequencing (Figure 1E). Thus, we confirmed that *SFT2D2‐TBX19* is composed of the first 8 exons of the parental *SFT2D2* gene and the last 7 exons of the parental *TBX19* gene. Our previous study has demonstrated that chimeric RNAs are mainly produced in the following three ways: gene fusion, trans‐splicing, and cis‐splicing.^[^ ^5^ ^]^ Since *SFT2D2* and *TBX19* are adjacent genes, we hypothesized that *SFT2D2‐TBX19* is formed by cis‐splicing. To substantiate this hypothesis, we further conducted cis‐splicing between adjacent genes (cis‐SAGe) validation experiments as previously reported.^[^ ^5^ ^]^ A specific oligonucleotide targeting exon 2 of *TBX19* was added into reverse transcription system instead of oligo‐dT to amplify all the transcripts that contain sequences of *TBX19*. The junction sequence of intron 7 and exon 8 of *SFT2D2* was amplified (Figure 1F,G), supporting the presence of a precursor mRNA that spans the connecting sequence between *SFT2D2* and *TBX19*. Therefore, chimeric *SFT2D2‐TBX19* is considered as a product of cis‐SAGe.

### 2.3 *SFT2D2‐TBX19* Enhances the Proliferation, Migration, and Invasion of Prostate Cancer Cells Both In Vivo and In Vitro

**Figure 2:**
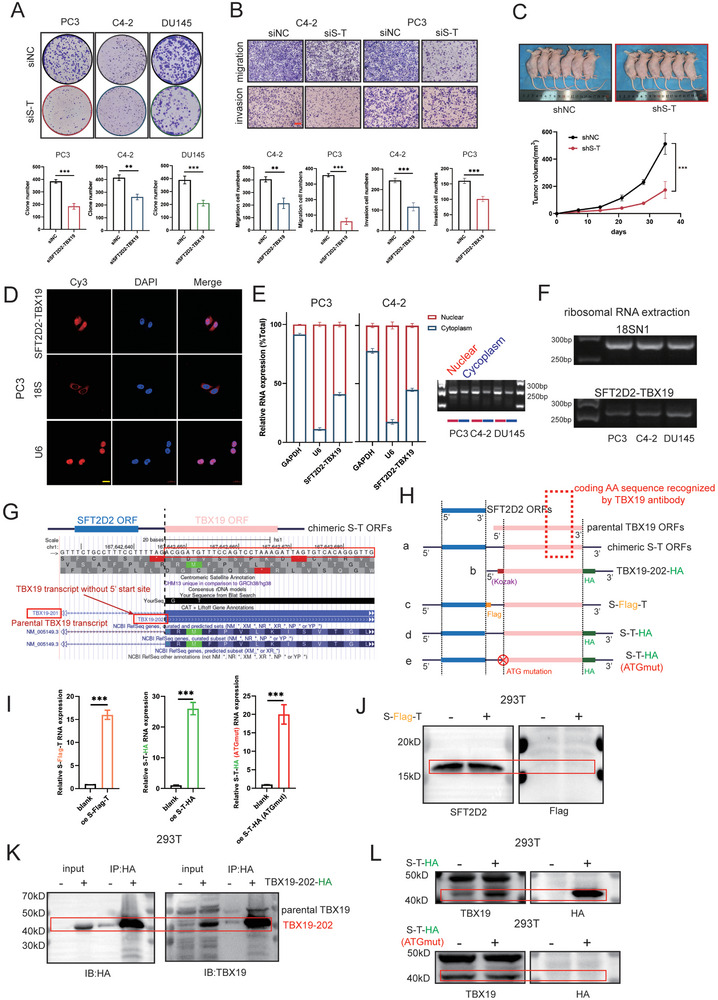
*SFT2D2‐TBX19* can encode protein TBX19‐202. A) Colony formation assays of PC3, C4‐2, DU145 cells in six‐well plates following *SFT2D2‐TBX19* knocked down. B) Migration and invasion assays of PC3, C4‐2 cells following *SFT2D2‐TBX19* knocked down. The scale bar in the lower left corner represents 200 µm. C) Subcutaneous tumor formation in nude mice after injecting 1 × 10^7^ shNC or sh*SFT2D2‐TBX19* PC3 cells. The tumor volume was measured weekly. D) RNA fluorescence in situ hybridization was performed for *SFT2D2‐TBX19*, with U6 and DAPI used as nucleus location references, and 18s as a cytoplasm location reference. The scale bar in the lower left corner represents 20 µm. E) RT‐qPCR analysis and agarose Gel confirmation of *SFT2D2‐TBX19* in nuclear and cytoplasm extracts, with U6 used as a nucleus location reference and GAPDH as a cytoplasm location reference. F) Agarose Gel confirmation of *SFT2D2‐TBX19* after PCR amplification following ribosomal RNA extraction, with 18SN1 used as a positive reference for ribosomal RNA. G) Results of comparing the full‐length sequence of *SFT2D2‐TBX19* on the UCSC website. H) Abridged overview of constructed plasmids. Plasmid (a) represents full length of *SFT2D2‐TBX19*. The speculated ORFs of *SFT2D2‐TBX19* and the encoding proteins of parental SFT2D2 and TBX19 are compared. Red dashed box represents identified amino acid sequence of TBX19 antibody. Plasmid (b) represents the speculated TBX19‐202. Plasmid (c) Flag tag sequences were added after the first ORF, which corresponds to parental SFT2D2. Plasmid (d) HA tag sequences were added after the second ORF, which corresponds to the speculated TBX19‐202. Plasmid (e) is the ATG mutation form of plasmid (d). I) RT‐qPCR assessment of RNA levels after transfecting plasmids S‐Flag‐T, *SFT2D2‐TBX19*‐HA and *SFT2D2‐TBX19*‐HA(ATGmut) into HEK293T cells. J) SFT2D2 and Flag blotting after transfecting plasmid S‐Flag‐T into HEK293T cells. Red wireframe shows the location of Flag‐labeled protein on the band. K) HA and TBX19 blotting after HA immunoprecipitation from HEK193T cells transfected with TBX19‐202‐HA. Red wireframe represents the location of HA‐labeled protein on the band. L) TBX19 and HA blotting were performed after transfecting HEK293T cells with plasmid *SFT2D2‐TBX19*‐HA or *SFT2D2‐TBX19*‐HA (ATGmut). Red wireframe shows the location of HA‐labeled protein on the band. Data are represented as mean ± SD. A,B,I: *n* = 3, Student's t test; C, *n* = 5, Student's t test, **p < 0.01, ***p < 0.001.

Our additional experiments indicated that *SFT2D2‐TBX19* overexpression promoted cell proliferation, migration and invasion (Figure 1H,I and Figure S1F, Supporting Information), revealing that *SFT2D2‐TBX19* may play an oncogenic role in prostate tumor development. Subsequently, we designed specific siRNA targeting the junction sequence of *SFT2D2‐TBX19*. The results indicated that si*SFT2D2‐TBX19* significantly interfered with the transcription of *SFT2D2‐TBX19*, without affecting the transcription of the parental genes *SFT2D2* or *TBX19* (Figure S2A, Supporting Information), or the protein levels (Figure S2B, Supporting Information). Importantly, *SFT2D2‐TBX19* knockdown inhibited cancer cell proliferation, colony formation, migration and invasion (**Figure** **2**A,B and Figure S2C–F, Supporting Information). The xenograft experiments also suggested that the sh*SFT2D2‐TBX19* group exhibited a smaller tumor volume (*p* < 0.05) (Figure 2C). These results supported that *SFT2D2‐TBX19* is a potential novel oncogene in prostate tumor development.

### 2.4 *SFT2D2‐TBX19* Encodes Protein TBX19‐202, Which Promotes Cell Proliferation, Migration, and Invasion

The distinct cellular localization of chimeric RNAs plays a crucial and varied role in regulating cellular metabolism. FISH (Figure 2D and Figure S3A, Supporting Information) and RT‐qPCR analysis of nuclear and cytoplasmic extracts (Figure 2E) revealed that *SFT2D2‐TBX19* was distributed in both the nucleus and the cytoplasm, suggesting its diverse functional roles. We predicted the open reading frames (ORFs) of *SFT2D2‐TBX19* using SnapGene, which identified two ORFs. The first predicted ORF was identical to the parental *SFT2D2*. Interestingly, the second predicted ORF matched TBX19‐202 on UCSC, although UCSC did not provide its 5′ UTR sequence (Figure 2G). On one hand, ribosome analysis indicated that *SFT2D2‐TBX19* might encode peptides, as evidenced by its amplification from ribosomes (Figure 2F). On the other hand, to further confirm whether *SFT2D2‐TBX19* can encode proteins, we designed several plasmids based on the two predicted ORF sequences (Figure 2H): a) the full length of *SFT2D2‐TBX19*; b) the speculated TBX19‐202‐HA; c) *SFT2D2*‐Flag‐*TBX19*, which includes Flag tag sequences added after the first ORF (matching the ORF of the parental *SFT2D2*); d) *SFT2D2‐TBX19*‐HA, with HA tag sequences added after the second ORF; and e) a start codon mutation version of *SFT2D2‐TBX19*‐HA (second ORF, loss of encoding ability). After overexpressing *SFT2D2‐*Flag*‐TBX19* in 293T cells (left panel, Figure 2I), the Flag‐conjugated agarose beads were used to immunoprecipitate the tagged proteins. The results showed no increase in SFT2D2 after transfection with the Flag‐tagged plasmid, and Flag was not detected at the expected band position (Figure 2J), suggesting that the first ORF of *SFT2D2‐TBX19* is not functional. The second ORF, which aligns with most of the parental TBX19 protein's amino acid sequence, was termed TBX19‐202 (Figure S3B, Supporting Information). HA‐conjugated agarose beads were used to immunoprecipitate the TBX19‐202‐HA peptides. The TBX19 antibody identified common peptides between TBX19 and TBX19‐202, and the HA antibody was used on the corresponding membrane simultaneously. As shown in Figure 2K, we confirmed that the TBX19 antibody could recognize both the parental TBX19 protein and the predicted TBX19‐202 peptides. Furthermore, after overexpressing *SFT2D2‐TBX19*‐HA (middle panel, Figure 2I), TBX19‐202 was significantly increased in the transfected group at the corresponding band position (upper panel, Figure 2L). After introducing an ATG mutation in *SFT2D2‐TBX19*‐HA and overexpressing it in 293T cells (right panel, Figure 2I), there was no increase in TBX19‐202, and HA was not detected (lower panel, Figure 2L). We further observed that both overexpression (oe*SFT2D2‐TBX19*) and knockdown (sh*SFT2D2‐TBX19*) of wild‐type *SFT2D2‐TBX19* regulated the expression levels of TBX19‐202 in PC3 and C4‐2 cells (Figure S3C, Supporting Information), confirming that *SFT2D2‐TBX19* encodes the second ORF, TBX19‐202, whose 5′UTR was identified in this study.

**Figure 3:**
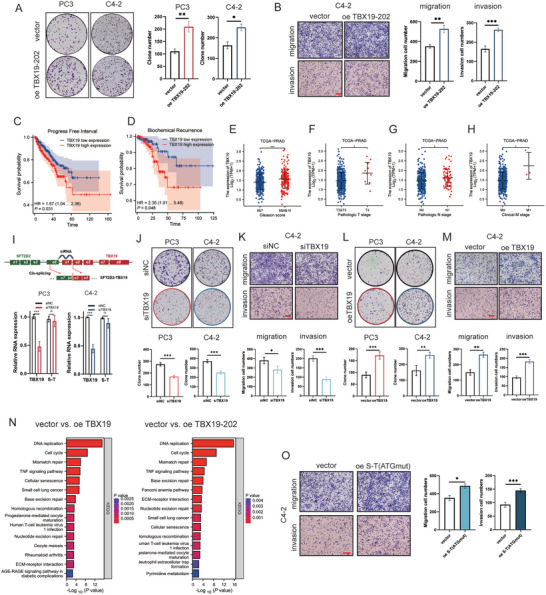
Parental TBX19 is closely associated with poor prognosis and promotes cell proliferation, migration and invasion in prostate cancer. A) Colony formation assays of PC3, C4‐2 cells after the overexpression of TBX19‐202. B) Migration and invasion assays of C4‐2 cells after the overexpression of TBX19‐202. The scale bar in the lower left corner represents 200 µm. C,D) The analysis of progress‐free interval and biochemical recurrence between TBX19 low and high expression groups from TCGA database. E) Analysis of TBX19 expression between Gleason score ≤7 and >7 from TCGA database. F–H) Analysis of TBX19 expression across different pathologic T stage, pathologic N stage and clinical stage from TCGA database. I) Schematic diagram of siRNA design for knocking down parental TBX19 and RT‐qPCR assessment of TBX19 knockdown. J,L) Colony formation assays of PC3 and C4‐2 cells after parental TBX19 knockdown and overexpression. K,M) Migration and invasion assays of C4‐2 cells when parental TBX19 was knocked down and overexpressed. The scale bar in the lower left corner represents 200 µm. N) KEGG signaling analysis in mutual different enrichment following parental TBX19 and TBX19‐202 overexpression in PC3 cells. O) Migration and invasion analysis of C4‐2 cells transfected with vector, and ATG mutated *SFT2D2‐TBX19* plasmid. The scale bar in the lower left corner represents 200 µm. Data are represented as mean ± SD. A,B,I–M,O: *n* = 3, Student's t test; C,D: Cox proportional‐hazards model analysis; E–H: Kruskal‐Wallis test. *p < 0.05, **p < 0.01, ***p < 0.001.

To explore the biological function of TBX19‐202, we constructed overexpressed cell systems in PC3 and C4‐2 cells by using the plasmid TBX19‐202‐HA (Figure S3D, Supporting Information). The results showed that TBX19‐202 promotes cell proliferation, migration and invasion (**Figures** **3**A,B and S3E–H, Supporting Information). Therefore, the oncogenic effect of *SFT2D2‐TBX19* is at least partially mediated through its encoded protein, TBX19‐202.

### 2.5 The Parental TBX19 Also Enhances the Development of Prostate Cancer

Given the high similarity in amino acid sequences between parental TBX19 and TBX19‐202, they are likely to have similar regulatory effects. We further observed that high expression level of *TBX19* was associated with lower survival probabilities during progression‐free intervals (Figure 3C) and biochemical recurrence in TCGA dataset (Figure 3D). Additionally, *TBX19* transcripts were more prevalent in cases with the Gleason score >7, pathologic T4 stage, pathologic N1 stage, and clinical M1 stage (Figure 3E–H). These findings suggest that *TBX19* may act as an oncogene in prostate cancer. To confirm this, we designed specific siRNA targeting exon 1 of parental *TBX19* to avoid affecting *SFT2D2‐TBX19* transcription, and assessed the knockdown efficiency (Figure 3I). Knockdown of parental *TBX19* inhibited prostate cancer cell proliferation, colony formation, migration and invasion (Figure 3J,K and Figure S4A–D, Supporting Information). Conversely, overexpression of parental *TBX19* promoted cell proliferation, colony formation, migration and invasion (Figure 3L,M and Figure S4E–J, Supporting Information) in PC3 and C4‐2 cells. Given their similar amino acid sequences, we speculated that TBX19‐202 and the parental TBX19 may regulate analogous downstream signaling pathways. Further RNA sequencing revealed that the top four molecular pathways were consistent: DNA replication, cell cycle, mismatch repair, and TNF signaling (Figure 3N). In summary, *SFT2D2‐TBX19* could encode TBX19‐202, which shares a similar oncogenic role with the parental TBX19 in promoting the progression of prostate cancer.

### 2.6 *SFT2D2‐TBX19* Also Functions as a LncRNA to Stimulate Prostate Cancer Development

Considering that the UTR region of *SFT2D2‐TBX19* shares a substantial portion of its nucleotide sequence with the parental gene *SFT2D2*, we investigated the potential role of *SFT2D2* lncRNA in prostate cancer progression. Initially, we found no correlation between the *SFT2D2* transcript and the survival probabilities of prostate cancer patients in CPGEA database (Figure S5A, Supporting Information). Subsequently, we developed an ATG‐mutated *SFT2D2* plasmid to eliminate the potential impact of SFT2D2 protein. Our experiments revealed that the overexpression of *SFT2D2* lncRNA did not impact the proliferation, migration and invasion of prostate cancer cells (Figure S5B–D, Supporting Information). This suggests that the molecular function of *SFT2D2‐TBX19* might not depend on the RNA sequence of *SFT2D2*.

Notably, when comparing the transcript levels of chimeric *SFT2D2‐TBX19* RNA and parental *TBX19* RNA using PCR standard amplification curves, the transcript number of parental *TBX19* was 10^2^–10^3^ times higher than that of chimeric *SFT2D2‐TBX19* in six prostate cancer cell lines (Figure S6A–F, Supporting Information). Given that the expression level of *SFT2D2‐TBX19* was significantly lower than that of *TBX19*, and *TBX19* itself was observed to promote prostate cancer development, we speculated that the *SFT2D2‐TBX19* lncRNA may possess distinct molecular functions beyond encoding TBX19‐202. To test this hypothesis, we established the overexpression systems for *SFT2D2‐TBX19* (ATGmut) in C4‐2 and PC3 cells. The results demonstrated that *SFT2D2‐TBX19* lncRNA could also promote the proliferation, migration, and invasion of prostate cancer cells (Figure 3O and Figure S6G–I, Supporting Information).

**Figure 4:**
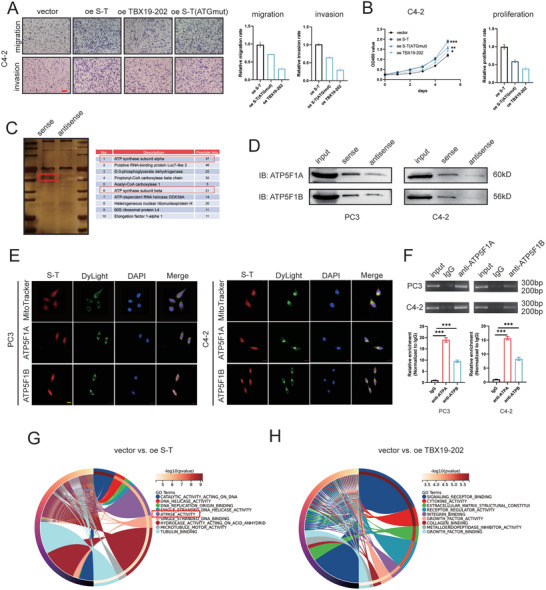
*SFT2D2‐TBX19* interacts ATP synthetase subunits ATP5F1A, ATP5F1B. A) Analysis of migration and invasion phenotypes of C4‐2 cells transfected separately with vector, wild‐type *SFT2D2‐TBX19*, ATG mutated *SFT2D2‐TBX19*, and TBX19‐202. The scale bar in the lower left corner represents 200 µm. B) Cell viability testing and proliferation rate analysis of C4‐2 cells transfected separately with vector, wild‐type *SFT2D2‐TBX19*, ATG mutated *SFT2D2‐TBX19*, and TBX19‐202. C) The image of silver staining of RNA pulldown with *SFT2D2‐TBX19* sense and antisense biotin‐labeled probes. The right table lists the top ten identified proteins from mass spectrometry sequencing. D) Western blot analysis of pulldown proteins from *SFT2D2‐TBX19* sense and antisense in PC3 and C4‐2 cells, immunoblotted with ATP5F1A and ATP5F1B antibodies. E) Immunofluorescence co‐localization of *SFT2D2‐TBX19* with mitochondria, ATP synthase F1 subunits ATP5F1A and ATP5F1B in PC3 and C4‐2 cells. The scale bar in the lower left corner represents 20 µm. F) RT‐qPCR and agarose gel electrophoresis analysis of *SFT2D2‐TBX19* amplification products immunoprecipitated by ATP5F1A and ATP5F1B antibodies. G,H) Go term analysis of molecular function enrichments for differentially expressed genes conducted through RNA sequence in PC3 cells following *SFT2D2‐TBX19* and TBX19‐202 overexpression. Data are represented as mean ± SD. B,F: *n* = 3, one‐way ANOVA with Fisher's LSD was used to determine statistical significance, *p < 0.05, **p < 0.01, ***p < 0.001.

To determine which attribute—lncRNA or TBX19‐202—plays a primary role in tumor cell development, we performed cell functional assays with equivalent TBX19‐202 translation between *SFT2D2‐TBX19* and TBX19‐202 overexpression, as well as equivalent *SFT2D2‐TBX19* lncRNA transcription between *SFT2D2‐TBX19* and its ATG‐mutated variant, *SFT2D2‐TBX19* (ATGmut) (Figure S7A,B, Supporting Information). The results indicated that *SFT2D2‐TBX19* lncRNA contributed approximately 60–70% to tumor cell development, while TBX19‐202 accounted for about 30–40% (**Figures** **4**A,B and S7C,D, Supporting Information). Given the significance of *SFT2D2‐TBX19* in its lncRNA form, it is crucial to further investigate its molecular mechanisms driving prostate cancer progression.

### 2.7 *SFT2D2‐TBX19* Interacts with F1 Subunits of Mitochondrial ATP Synthase

To elucidate the lncRNA mechanisms of *SFT2D2‐TBX19*, we performed RNA pulldown assays followed by silver staining to differentiate the proteins pulled down from the sense and antisense groups. Mass spectrometry analysis revealed that the primary enriched proteins were the subunits ATP5F1A and ATP5F1B of the mitochondrial ATP synthase (Figure 4C and Table S4, Supporting Information). Western blot analysis further verified the presence of these proteins in both the sense and antisense pulldown samples from PC3 and C4‐2 cells (Figure 4D). Immunofluorescence imaging confirmed that *SFT2D2‐TBX19* colocalized with ATP5F1A and ATP5F1B, as well as with mitochondria structures (Figure 4E). Subsequent RNA immunoprecipitation assays using ATP5F1A and ATP5F1B antibodies further confirmed the interaction between *SFT2D2‐TBX19* and these two mitochondrial ATP synthase subunits (Figure 4F).

Mitochondrial membrane ATP synthase, also known as F1F0 ATP synthase or Complex V, generates ATP from ADP by exploiting a proton gradient across the membrane, which is created by the electron transport complexes of the respiratory chain. The ATP5F1A and ATP5F1B subunits constitute the catalytic core of the F1 complex. The rotation of the central stalk relative to the surrounding ATP5F1A_3_ATP5F1B_3_ subunits leads to hydrolysis of ATP.^[^ ^14^, ^15^ ^]^ Numerous studies have demonstrated that ATP synthase can influence prostate cancer development through ATP production.^[^ ^16^, ^17^ ^]^ Further RNA sequencing analysis demonstrated that the regulation of ATPase activity was enriched in *SFT2D2‐TBX19* overexpression group, but not in the TBX19‐202 overexpression group (Figure 4G,H). Further coimmunoprecipitation assays revealed that there was no interaction between the TBX19‐202 protein and ATP5F1A/ATP5F1B (Figure S8A, Supporting Information). These results suggested that *SFT2D2‐TBX19*, as a long non‐coding RNA, may mediate ATP synthase activity to regulate cell proliferation, migration and invasion in prostate cancer.

### 2.8 *SFT2D2‐TBX19* Augments ATP Synthase Enzyme Activity and ATP Production by Stabilizing the Interaction between ATP5F1A and ATP5F1B, Mediated through the Phosphorylation of ATP5F1A by TNK2/ACK1

**Figure 5:**
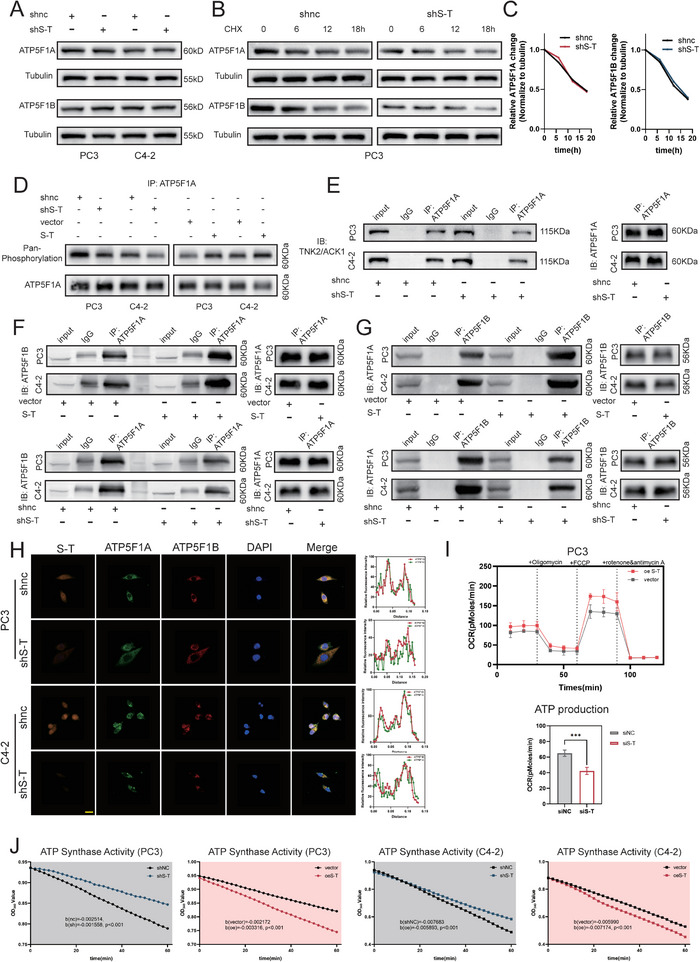
*SFT2D2‐TBX19* enhances ATP5F1A phosphorylation via TNK2/ACK1, stabilizing ATP synthase and increasing ATP production. A) Western blot analysis of ATP5F1A and ATP5F1B in PC3 and C4‐2 cells following *SFT2D2‐TBX19* knockdown. Beta‐tubulin serves as an internal reference protein. B,C) Analysis of ATP5F1A and ATP5F1B protein degradation after treatment with 50 µg mL^−1^ cycloheximide in PC3 cells following *SFT2D2‐TBX19* knockdown. Beta‐tubulin serves as an internal reference protein. D) Analysis of ATP5F1A phosphorylation in PC3 and C4‐2 cells following *SFT2D2‐TBX19* knockdown or overexpression, using the same immunoprecipitation quantity of ATP5F1A. E) Analysis of phosphokinase TNK2/ACK1 immunoprecipitation in PC3 and C4‐2 cells following *SFT2D2‐TBX19* knockdown, using the same immunoprecipitation quantity of ATP5F1A. F,G) The immunoprecipitation quantity of ATP5F1B or ATP5F1A in PC3 and C4‐2 cells after *SFT2D2‐TBX19* overexpression and knockdown. The same immunoprecipitation of ATP5F1A or ATP5F1B serves as an interval reference. H) Immunofluorescence co‐localization of ATP synthase F1 subunits ATP5F1A and ATP5F1B in PC3 and C4‐2 cells after *SFT2D2‐TBX19* knockdown. The scale bar in the lower left corner represents 20 µm. I) Analysis of mitochondrial oxygen consumption and ATP production in PC3 cells after *SFT2D2‐TBX19* knockdown. Data are represented as mean ± SD (*n* = 5 replicates). Student's t test was used to determine statistical significance, ***p < 0.001. J) Analysis of ATP synthase enzymatic activity in PC3 and C4‐2 cells after *SFT2D2‐TBX19* knockdown and overexpression. Univariate analysis using a general linear model was employed to compare the regression curves.

To explore whether *SFT2D2‐TBX19* regulates ATP production via ATP5F1A/B, we first conducted western blot analyses, which revealed that *SFT2D2‐TBX19* did not affect the protein levels of ATP5F1A or ATP5F1B (**Figure** **5**A). Subsequent experiments further showed that *SFT2D2‐TBX19* did not mediate ATP5F1A/B degradation via post‐translational ubiquitination (Figure 5B,C and Figure S8B,C, Supporting Information). Therefore, *SFT2D2‐TBX19* may not affect the production or degradation of ATP5F1A and ATP5F1B.

Previous research has revealed that ATP5F1A phosphorylation plays a crucial role in the interaction between ATP5F1A and ATP5F1B, as well as in ATP synthase activity and the protection of mitochondrial vulnerability.^[^ ^17^ ^]^ Building on these findings, we hypothesized that *SFT2D2‐TBX19* might regulate ATP synthase activity and protect mitochondrial function by modulating ATP5F1A phosphorylation. Further co‐IP validated that ATP5F1A phosphorylation was elevated when *SFT2D2‐TBX19* was overexpressed, and decreased vice versa (Figure 5D). TNK2/ACK1 is the only known kinase for ATP5F1A, and its immunoprecipitation decreased following *SFT2D2‐TBX19* knockdown (Figure 5E), suggesting that *SFT2D2‐TBX19* promotes ATP5F1A phosphorylation via TNK2/ACK1. To determine whether *SFT2D2‐TBX19* regulates the interaction between ATP5F1A and ATP5F1B, we measured the levels of ATP5F1A and ATP5F1B under identical immunoprecipitation conditions after *SFT2D2‐TBX19* knockdown or overexpression. The results indicated that *SFT2D2‐TBX19* facilitated a more stable interaction between ATP5F1A and ATP5F1B, while its knockdown disrupted this stability (Figure 5F–H). Further experiments showed that the stability of the ATP5F1A and ATP5F1B complex affected ATP production and ATP synthase enzyme activity (Figure 5I–J). These findings suggest that *SFT2D2‐TBX19* regulates ATP production by modulating the stability of the core F1 ATP5F1A and ATP5F1B complex.

**Figure 6:**
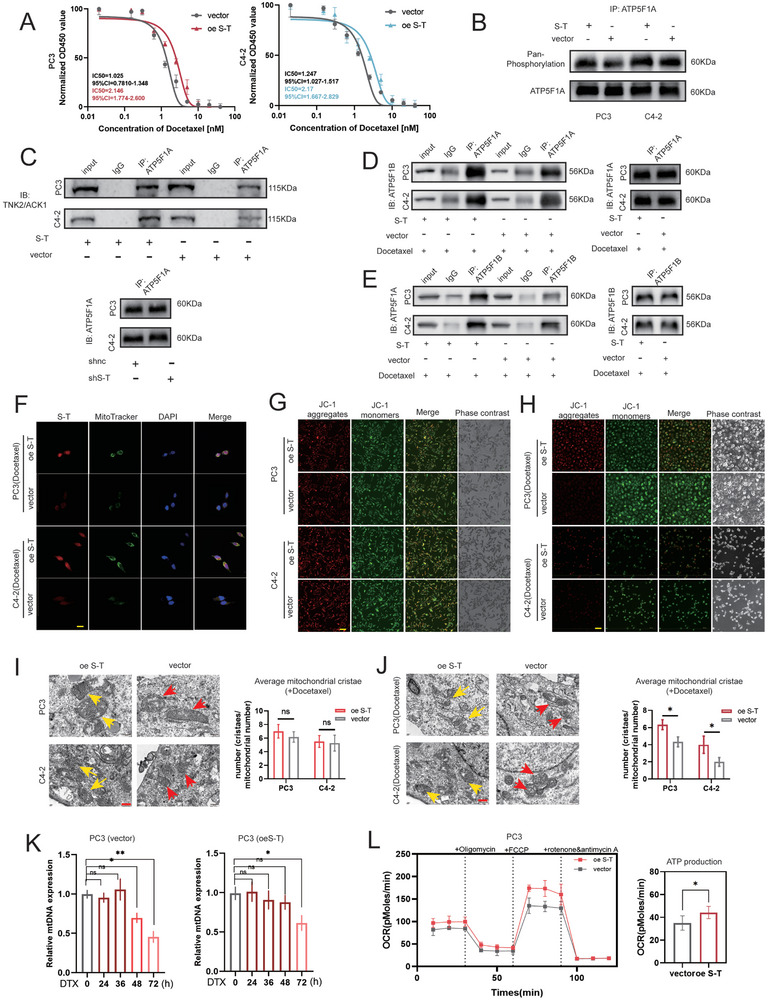
*SFT2D2‐TBX19* protects mitochondrial functions and structures via ATP5F1A phosphorylation to maintain cell proliferation after docetaxel treatment. A) IC50 analysis in PC3 and C4‐2 cells with *SFT2D2‐TBX19* overexpression after docetaxel treatment. B) Analysis of ATP5F1A phosphorylation in PC3 and C4‐2 cells with *SFT2D2‐TBX19* overexpression after docetaxel treatment, using the same immunoprecipitation quantity of ATP5F1A. C) Analysis of phosphokinase TNK2/ACK1 immunoprecipitation in PC3 and C4‐2 cells with *SFT2D2‐TBX19* overexpression after docetaxel treatment, using the same immunoprecipitation quantity of ATP5F1A. D,E) The immunoprecipitation quantity of ATP5F1B or ATP5F1A in PC3 and C4‐2 cells with *SFT2D2‐TBX19* overexpression after docetaxel treatment. The same immunoprecipitation of ATP5F1A or ATP5F1B serves as an interval reference. F) Immunofluorescence co‐localization of ATP5F1A and ATP5F1B in PC3 and C4‐2 cells with *SFT2D2‐TBX19* overexpression after docetaxel treatment. The scale bar in the lower left corner represents 20 µm. G,H) Analysis of mitochondrial membrane potential in PC3 and C4‐2 cells with *SFT2D2‐TBX19* overexpression, both before and after docetaxel treatment. The scale bar in the lower left corner represents 100 µm. I,J) Ultrastructural morphology of mitochondria in PC3 and C4‐2 cells with *SFT2D2‐TBX19* overexpression, both before and after docetaxel treatment. The scale bar in the lower left corner represents 500 nm. K) RT‐qPCR analysis of mtDNA and HBB levels in PC3 cells with *SFT2D2‐TBX19* overexpression treated with 4 × 10^−9^ m of docetaxel for 0, 24, 36, 48, and 72 h. HBB serves as an internal control gene. L) Analysis of mitochondrial stress and ATP production in PC3 cells with *SFT2D2‐TBX19* overexpression after docetaxel treatment. Data are represented as mean ± SD. I,J: *n* = 2, Student's t test; K: *n* = 3, one‐way ANOVA with Fisher's LSD; L: *n* = 5, Student's t test, *p < 0.05, **p < 0.01, ***p < 0.001.

Next, we constructed a model of mitochondrial damage induced by docetaxel^[^ ^18^, ^19^, ^20^, ^21^ ^]^ (Figure S8D–F, Supporting Information). Our studies revealed that *SFT2D2‐TBX19* continued to support prostate cancer cell proliferation even after docetaxel treatment (**Figure** **6**A). It also maintained the ability to enhance ATP5F1A phosphorylation through TNK2/ACK1 (Figure 6B,C). *SFT2D2‐TBX19* overexpression stabilized the interaction between ATP5F1A and ATP5F1B even after docetaxel treatment (Figure 6D–F). In the absence of docetaxel, there were no significant changes in mitochondrial membrane potential or structures in PC3 or C4‐2 cells with stable *SFT2D2‐TBX19* overexpression (Figure 6G,I). However, *SFT2D2‐TBX19* significantly protected mitochondrial membrane potential and preserved normal mitochondrial structures after docetaxel treatment (Figure 6H,J,K). This protection helped to sustain ATP production, thereby promoting cell proliferation (Figure 6L). In summary, *SFT2D2‐TBX19* stabilizes the interaction between ATP5F1A and ATP5F1B through ATP5F1A phosphorylation, leading to increased ATP synthase activity and more ATP production. Additionally, it protects mitochondrial metabolism and structures under mitochondrial vulnerability.

### 2.9 The 1801–2400 bp Region of *SFT2D2‐TBX19* and the Intermediate Structural Domain of ATP5F1A Are Components Involved in Molecular Functional Processes

**Figure 7:**
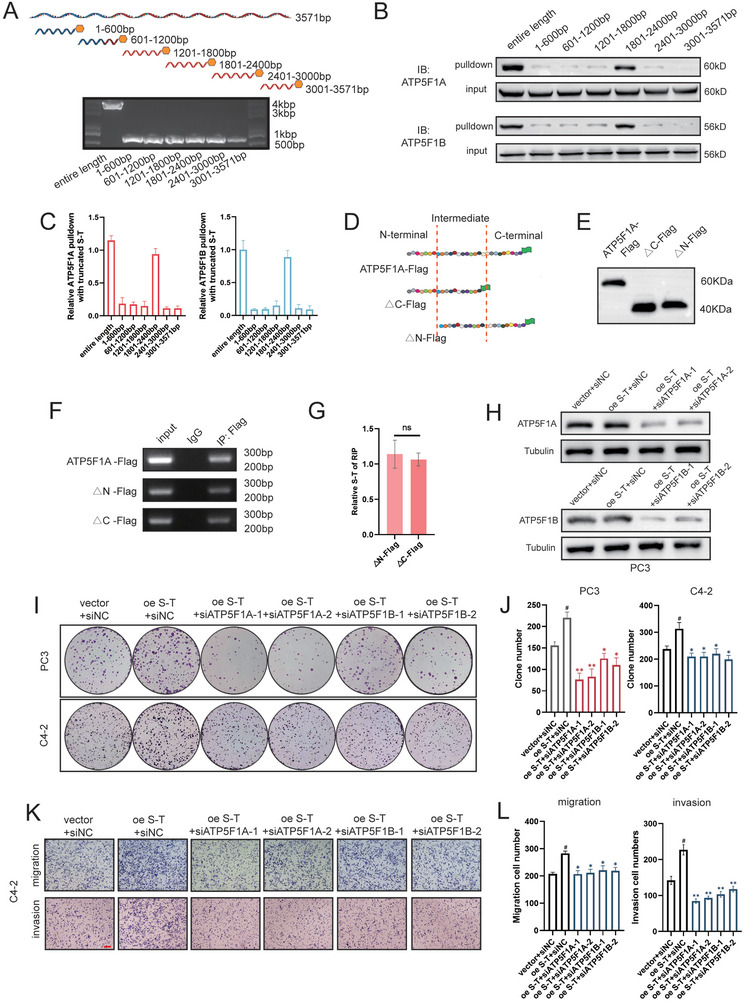
The 1801‐2400bp region of *SFT2D2‐TBX19* and the intermediate structural domain of ATP5F1A are key components of molecular functionality. A) Truncation of *SFT2D2‐TBX19* and its identification on an agarose gel after biotin‐labeled amplification. B,C) ATP5F1A/B pulldown and quantitative analysis using truncated biotin‐labeled RNA probes. D,E) Structural domain division of ATP5F1A and its validation using Western blot. F,G) RIP and statistical analysis of immunoprecipitation using Flag antibodies. H) Validation of ATP5F1A and ATP5F1B protein levels in PC3 cells with *SFT2D2‐TBX19* overexpression after ATP5F1A or ATP5F1B knockdown. I,J) Colony formation and statistical analysis in PC3 and C4‐2 cells with *SFT2D2‐TBX19* overexpression after ATP5F1A or ATP5F1B knockdown. K,L) Migration and invasion assays of C4‐2 cells with *SFT2D2‐TBX19* overexpression after ATP5F1A or ATP5F1B knockdown. Data are represented as mean ± SD. G: *n* = 3, Student's t test; J,L: *n* = 3, one‐way ANOVA with Fisher's LSD. ns‐ no significant difference, *p < 0.05, **p < 0.01‐compared with oe *SFT2D2‐TBX19*+siNC; ^#^ *p* < 0.05‐compared with vector+siNC.

To investigate the functional regions between *SFT2D2‐TBX19* and ATP5F1A, truncated versions of *SFT2D2‐TBX19*, each 600 bp long, were synthesized and validated by gel electrophoresis (**Figure** **7**A). Pulldown assays with truncated *SFT2D2‐TBX19* revealed that the 1801–2400 bp region was the most significant RNA sequence of *SFT2D2‐TBX19* for binding ATP5F1A and ATP5F1B (Figure 7B,C). The *TBX19* transcript also contains the 1801–2400 bp region of *SFT2D2‐TBX19*. To investigate the association between ATP5F1A/B and *TBX19* transcript, we performed RNA pulldown assays and found that *TBX19* transcript could also interact with ATP5F1A/B. However, under the condition of equal mol quantity of probes, *SFT2D2‐TBX19* lncRNA interacted with more ATP5F1A/B, suggesting that *SFT2D2‐TBX19* has a stronger binding affinity (Figure S8G, Supporting Information). Furthermore, after extracting and purifying mitochondrial RNAs, we found that the number of *SFT2D2‐TBX19* transcripts in the mitochondria was approximately 10^2^ times higher than that of *TBX19* transcripts in the mitochondria even though the overall abundance of *SFT2D2‐TBX19* is much lower than that of *TBX19* (Figure S8H, Supporting Information). This confirms that *TBX19* lacks the ability to translocate into mitochondria. Importantly, we did not find evidence that *TBX19* affected the activity of ATP synthase (Figure S8I, Supporting Information). In summary, due to its low protein‐binding ability of *TBX19* and the difficulty entering mitochondria, *TBX19* lacks the capacity to regulate mitochondrial ATP synthesis through interaction with ATP5F1A and ATP5F1B, as observed with *SFT2D2‐TBX19*.

Given that *SFT2D2‐TBX19* regulates ATP5F1A phosphorylation, we designed and validated two structural domains of ATP5F1A, △N‐Flag and △C‐Flag, for RNA immunoprecipitation (Figure 7D,E). The amount of *SFT2D2‐TBX19* immunoprecipitated by △N‐Flag and △C‐Flag indicated that the intermediate structural domain of ATP5F1A interacted with *SFT2D2‐TBX19* (Figure 7F,G). Additionally, we performed rescue cell phenotype experiments. Knockdown of ATP5F1A and ATP5F1B was confirmed by Western blot after siRNA transfection (Figure 7H, Figure S9A, Supporting Information). Prostate cancer cell proliferation, migration and invasion were reversed in the *SFT2D2‐TBX19* overexpression group following ATP5F1A or ATP5F1B knockdown (Figure 7I–L and Figure S9B–E, Supporting Information). These findings further suggest that the interaction between ATP5F1A and ATP5F1B is a key molecular signal mediated by *SFT2D2‐TBX19*.

## 3 Discussion

**Figure 8:**
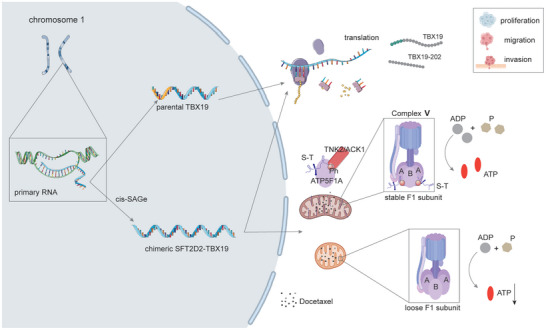
*SFT2D2‐TBX19* facilitates prostate cancer progression by encoding TBX19‐202 protein and stabilizing mitochondrial ATP synthase through ATP5F1A phosphorylation.

In this study, we identified an oncogenic chimeric *SFT2D2‐TBX19* in prostate cancer. This research clarified the complete 5′UTR sequence of TBX19‐202 encoded by the chimeric *SFT2D2‐TBX19*. Both the parental TBX19 and the TBX19‐202 peptides contribute to prostate cancer progression through molecular pathways such as DNA duplication, cell cycle, and TNF signaling. *SFT2D2‐TBX19* can also work as a lncRNA, enhancing ATP synthase F1 subunit ATP5F1A phosphorylation via TNK2/ACK1, thereby stabilizing its interaction with ATP5F1B. This stable interaction protects the mitochondrial structures and supports ATP production, which in turn promotes prostate cancer cell proliferation, migration and invasion (**Figure** **8**).

Approximately 75% of the human genome is transcribed, with about 98% of these transcripts being long noncoding RNAs (lncRNAs).^[^ ^22^ ^]^ Generally, lncRNAs are defined as RNA transcripts longer than 200 nucleotides that lack protein‐coding potential.^[^ ^23^ ^]^ However, increasing evidences have revealed that some lncRNAs can encode peptides.^[^ ^24^ ^]^ Research has clarified this potential: exons of coding lncRNAs are often more highly conserved than those of protein‐coding genes, enabling the identification of open reading frames (ORFs) and translation regulatory regions.^[^ ^24^, ^25^ ^]^ This provides the fundamental RNA sequences necessary for encoding. Additionally, an important criterion is that cytoplasmic lncRNAs are directly associated with ribosomes, the sites of peptide synthesis.^[^ ^26^ ^]^ The essence of encoding involves specific RNA frames being translated into proteins by ribosomes. In recent years, the potential of lncRNAs to encode short or long peptides has been increasingly recognized.^[^ ^27^, ^28^, ^29^, ^30^ ^]^ As our previous study reported, *TMPRSS2‐ERG* e1e4 isoform and *TMPRSS2‐ERG* e2e4 isoform have distinctly different biological functions, even though e1e4 isoform is only 31 amino acids shorter than e2e4 isoform.^[^ ^9^ ^]^ This suggests that changes in peptide abundance can assign new functions and roles in cell metabolism and molecular signaling. A detailed differential analysis of the biological and molecular characteristics between TBX19‐202 and parental TBX19 should be addressed in future research.

Non‐coding RNAs typically mediate interactions between proteins, post‐transcriptional modifications, protein ubiquitination, and phosphorylation,^[^ ^31^, ^32^, ^33^, ^34^ ^]^ among other processes. Mitochondria are reported essential for regulating redox homeostasis, oncogenic signaling, innate immunity, and apoptosis.^[^ ^35^ ^]^ Our study indicated that *SFT2D2‐TBX19* does not regulate the common degradation of mitochondrial ATP synthase F1 subunits ATP5F1A or ATP5F1B. However, ATP5F1A phosphorylation can be regulated to mediate the biological activity of ATP synthase.^[^ ^17^ ^]^ Our research confirmed that *SFT2D2‐TBX19* regulates ATP5F1A phosphorylation, thereby enhancing the stable interaction between ATP5F1A and ATP5F1B. The exact phosphorylation sites still required further exploration. Furthermore, docetaxel‐based chemotherapy for prostate cancer continues to face significant challenges.^[^ ^36^ ^]^ Numerous studies have demonstrated that mitochondrial metabolism plays an indispensable role in the susceptibility of cancer cells to docetaxel.^[^ ^37^, ^38^ ^]^ *SFT2D2‐TBX19* plays a protective role in mitochondrial metabolism, preserving mitochondrial membrane potential and normal structures in the harsh environment induced by docetaxel, thereby promoting cell survival in prostate cancer. The analysis of truncation of *SFT2D2‐TBX19* and structural domain of ATP5F1A offers insights into its potential as a target for chemotherapy in prostate cancer.

*SFT2D2‐TBX19* exhibits a greater ability to enter mitochondria compared to *TBX19* transcript. We hypothesize that certain specific RNA secondary or tertiary structures of *SFT2D2‐TBX19* facilitate its translocation to the mitochondria. This process may require mitochondrial translocation protein factors including glycolytic enzyme enolase (ENO2P), lysyl‐tRNA synthetase (preMSK or pre‐LysRS), and the translocase TOM/TIM complex.^[^ ^39^, ^40^ ^]^ But the specific protein factors in this process still need to be explored.

Chimeric RNAs are considered as products of the evolutionary history of the human genome. Some chimeric RNAs may assume functions that differ from those of their parental genes, as observed with *HNRNPA1L2‐SUGT1* and e2e4 *TMPRSS2‐ERG*.^[^ ^9^, ^41^ ^]^ Moreover, novel chimeric RNAs continue to emerge and evolve in tumor development. It is probable that *SFT2D2‐TBX19* and TBX19‐202 gradually replace the biological functions of their parental genes and TBX19 protein.

NEPC, an aggressive subtype evolved from CRPC, is typically resistant to nearly all current therapies.^[^ ^42^, ^43^ ^]^ We found that *SFT2D2‐TBX19* was gradually increased from castration‐resistant prostate cancer cell lines PC3, C4‐2, DU145, 22RV1 to neuroendocrine prostate cancer cell lines NCI‐H660. The correlation heat map showed that *SFT2D2‐TBX19* was associated with NEPC markers CHGA and NCAM1 in CPGEA database Figure S9F, Supporting Information). In addition, TBX19‐202 was associated with well‐known oncogenic DNA replication, cell cycle, TNF signaling, which is similar to many oncogenes. The well‐known tumor suppressor genes *p53*, *pten*, *rb1* play a groundbreaking role in cell cycle regulation, cell proliferation, DNA replication. Moreover, other studies discovered that *p53*, *pten*, *rb1* triple‐knockout mice (PBCre4:*pten* ^f/f^:*rb1* ^f/f^:*p53* ^f/f^) presented an aggressive metastases in bone, liver and lung. This TKO tumor exhibited increased levels of the NEPC markers CHGA, SYP, and NSE.^[^ ^44^ ^]^ We assessed the expression of *p53*, *pten*, *rb1* in PC3 and C4‐2 cells with *SFT2D2‐TBX19* knockdown and overexpression. The results showed that *pten* was regulated by *SFT2D2‐TBX19* (Figure S9G, Supporting Information). Those data suggested that *SFT2D2‐TBX19* maybe mediate the neuroendocrine process of prostate cancer.

## 4 Experimental Section

### Cell Culture and Treatment

Prostate cancer cell lines 22RV1, PC3, DU145, C4‐2, PC3 M, and the human embryonic kidney cell line HEK293T were purchased from the American Type Culture Collection (ATCC). 22RV1, PC3, C4‐2, DU145, and PC3 M were cultured in RPMI‐1640 medium (Procell, Wuhan, China) supplemented with 10% fetal bovine serum (HyCyte, Suzhou, China) and 1% penicillin/streptomycin (NCMbio, Suzhou, China). In contrast, HEK293T were cultured in complete DMEM medium.

According to the Cell Counting Kit‐8 assay (APExBIO, USA) protocol, diluted reagent following required concentration was added to 96‐well microplate to detect cell viability on Tecan SPARK microplate reader (Research Triangle Park, NC, USA). 1000 or 1200 cells were applied to cell proliferation cck‐8 assessment and colony formation assay. In cell migration transwell assay, 80000 PC3, C4‐2, or 40000 DU145 cells (stable knockdown or overexpression and transient interference) diluted with 200 µL FBS‐free medium were seeded into upper insert chambers (8 µm pore size; JETBIOFIL, China), while 800µl complete medium was in the lower chamber. For cell invasion assay, diluted matrix adhesive (Yeasen, China) was applied on the bottom of the upper transwell insert filter chambers and incubated at 37 °C for 3–4 h. The same number of cells as used in the transwell assay were added to the upper chambers.

### RNA Extraction, Touchdown PCR, Sanger Sequence, and RT‐qPCR

According to the manufacturer's protocol for Trizol (Vazyme, China), cell lysates from 2 × 10^6^–5 × 10^6^ cells were dissociated with 1 mL of Trizol, vigorously vortexed, and incubated at room temperature for 10 min. Subsequently, 200 µL of chloroform was added, and the upper aqueous phase was collected. After isopropanol precipitation with an equal volume and washing with 75% ethanol, the purified RNA was fully dissolved in 50–100 µL of RNase‐free water. RNA concentration was measured using a Nanodrop One Spectrophotometer (Thermo Fisher, USA).^[^ ^13^ ^]^

For cDNA synthesis, 1 µg of fresh RNA was reverse‐transcribed using the HiScript III 1st Strand cDNA Synthesis Kit (+gDNA wiper) (Vazyme, China). Touchdown PCR was performed using Phanta High‐Fidelity DNA Polymerase (Vazyme, China). 0.7% agarose gel was prepared by mixing agarose (Biosharp, China) powder with 1 × TAE buffer (Tris, acetate, and EDTA solution) in a microwavable flask, and the amplification products were separated by electrophoresed at 120 V for 30 min to 1 h. The amplified DNA products were analyzed by agarose gel electrophoresis and visualized using a UV analyzer (TANON, China). Bands of interest were excised and purified using the FastPure Gel DNA Extraction Mini Kit (Vazyme, China). The purified DNA was then sequenced by Tsingke Biotechnology, Beijing, China.

For RT‐qPCR analysis, fresh RNA was reverse‐transcribed using the HiScript II Q RT SuperMix for qPCR (+g DNA wiper) (Vazyme, China) to synthesize the first‐strand cDNA. A 20 µL amplification system was prepared using RT‐qPCR SYBR Green Master Mix (Vazyme, China), 10 × 10^−6^ m forward and reverse primers, and nuclease‐free H_2_O. Quantitative PCR was performed on a QuantStudio 6 system (Applied Biosystems, USA) to monitor double‐stranded DNA amplification. mRNA and chimeric RNA expression levels were normalized to the internal control RNA GAPDH. All Primer sequences are listed in Tables S1 and S2 (Supporting Information).

### Nuclear and Cytoplasmic Extraction

Nuclear and cytoplasmic extracts were prepared using the NE‐PERTM Nuclear and Cytoplasmic Extraction Reagents Kit (Thermo Fisher, USA). Phenol‐chloroform extraction and glycogen precipitation were employed to isolate inner RIP RNA. RNA was then reverse‐transcribed and analyzed by RT‐qPCR to detect the transcription level of *SFT2D2‐TBX19*.

### Ribosome Extraction

Ribosomes were extracted according to Ribosome Extraction Kit's protocol (Bestbio, China). RNA was extracted from ribosomal pellet using the Trizol extraction method.

### Western Blot

1 × 10^6^ cells were lysed with 200–300 µL RIPA lysis buffer (APExBIO, USA) with protease and phosphatase inhibitor cocktail (NCMbio, China). After incubation at 100 °C for 5–10 min, 20 µg protein sample was loaded onto sodium dodecyl sulfate‐polyacrylamide gel electrophoresis (SDS‐PAGE) (EpiZyme, China) gel. The separated proteins were transferred onto a 0.45 µm PVDF membrane (Millipore, USA) at 250–300 mA for 90–120 min.^[^ ^45^ ^]^ After block with 5% bovine serum albumin (BSA; Biofroxx, Germany) solution in TBST buffer about 1 h, specific primary antibody (SFT2D2 (Immunoway, USA); TBX19 (Boiss, China); Flag (Proteintech, China); HA (Cell Signaling Technology, USA); ATP5F1A (Proteintech, China); ATP5F1B (Proteintech, China); Pan‐phosphorylation (Santa Cruz Biotechnology, USA); ANK2 (Immunoway, USA); beta‐tubulin or GAPDH (Proteintech, China)) were incubated overnight at 4 °C. Subsequently, the membranes were incubated with the corresponding goat anti‐mouse or rabbit HRP‐conjugated Affinipure IgG(H+L) (Proteintech, China) solution in 5% BSA. After three rinses in TBST buffer, the membranes were visualized using an imaging system GelView 6000 Pro (BLT, China). The protein expression was normalized to housekeeping controls beta‐tubulin or GAPDH.

### Co‐Immunoprecipitation

1×10^7^ cells were scraped and lysed in EBC buffer (50 × 10^−3^ m Tris‐HCl, 120 × 10^−3^ m NaCl, 0.5% v/v NP‐40) with protease and phosphatase inhibitor cocktails. The mixture was then rotated and incubated overnight at 4 °C. After centrifugation at 4 °C, the supernatant was incubated with primary antibody at 4 °C for 6 h. Protein A/G agarose beads (25 µL/1 mL lysate; Santa Cruz Biotechnology, USA) were added to lysates. After a 4 h incubation, the mixture was centrifuged at 4 °C, the proteins‐agarose pellet was suspended and washed third with cold NETN buffer (20 × 10^−3^ m Tris‐HCl, 100 × 10^−3^ m NaCl, 0.5% v/v NP‐40, 1 × 10^−3^ m EDTA). Centrifugate at 4 °C, loading buffer was added to the pellet, and purified proteins were detected by Western blotting.

### RNA Fluorescence In Situ Hybridization (FISH) and Immunofluorescence (IF)

FISH was performed to detect the localization of chimeric RNA *SFT2D2‐TBX19* and Mitotracker (Beyotime, China) in PC3 and C4‐2 cells. The FISH probe sequence for *SFT2D2‐TBX19* sequence is ctggaaacatccgtctcactccatgg (5′‐3′ sense). IF was used to assess the colocalization and difference among *SFT2D2‐TBX19*, ATP5F1A and ATP5F1B. The details can be found in the previous experiment protocols.^[^ ^46^ ^]^

### RNA Pulldown and Mass Spectrometry

*SFT2D2‐TBX19* biotin‐labeled sense and antisense probes were synthesized by using the T7 High Yield RNA Transcription Kit (Vazyme, China) and Pierce RNA 3′ End Desthiobiotinylation Kit (Thermo Fisher, USA). The RNA pulldown assay was performed according to the protocol provided by the RNA Pulldown Kit (BersinBio, China). Pulled down proteins were separated using Western blot. Protein Stains K (Sangon, China) was used to detect the different pulldown protein bands. The SDS‐PAGE bands were excised and analyzed by mass spectrometry, which was conducted by Wininnovate Bio (Shenzhen, China). Primer sequences required for the biotin probes are listed in Table S2 (Supporting Information).

### RNA Immunoprecipitation (RIP) Assays

Approximately 2 × 10^7^ PC3, C4‐2, HEK293T cells were digested, washed in PBS buffer solution. After fixation in 3% polyformaldehyde and hybridization in glycine solution, the cell pellet was lysed in lysis buffer (50 × 10^−3^ m Tris‐HCl, 10 × 10^−3^ m EDTA, 1%SDS) with PMSF, proteinase inhibitor cocktail and RNase inhibitor. The mixture was then quickly subjected to freeze‐thaw cycles and centrifuged to separate the supernatant into input, IgG, and RIP groups. RIP group was incubated with 4 µg anti‐ATP5F1A, ATP5F1B antibody to immunoprecipitate the ATP5F1A or ATP5F1B‐RNA complexes, while IgG group was incubated with IgG as a negative control. After incubation of antibody, cell lysate, protein A/G magnetic beads for 4 hours at 4 °C, RNA Proteinase K buffer pH7.0 (100 × 10^−3^ m NaCl, 10 × 10^−3^ m Tris‐HCl pH7.0, 1 × 10^−3^ m EDTA pH8.0, 0.5% SDS) and proteinase K (Beyotime, China) were used to digest inner target proteins and antibodies. Phenol chloroform and glycogen precipitation extracted inner RIP RNA. RNA reverse transcription and RT‐qPCR were detected *SFT2D2‐TBX19* transcription level.

### siRNA Transfection

siRNA sequences were all provided in Table S3 (Supporting Information). 4 µL 20 × 10^−6^ m siRNA, 4 µL lipofectamine RNA iMAX (Invitrogen, USA) were mixed and incubated in 250 µL opti‐MEM (Gibco, USA). Subsequently, the mixture was added into 60–70% confluent cells with 750 µL penicillin/ streptomycin free RPMI‐1640. The medium was replaced with fresh complete medium after about 8 h. After 36–72 h, cell phenotype experiments were conducted.

### Transfection and Lentiviral Infection

Plasmids were transfected into PC3, C4‐2 cells according to the protocol provided by Lipofectamine 3000 (Invitrogen, China). Briefly, 4 µL lipofectamine 3000 was diluted in 125 µL opti‐MEM and 4 µL P3000, 2 µg plasmid were diluted in 125 µL opti‐MEM. After mixing and incubating about 15 min, 250 µL mixture was added into 2 mL medium in six‐well plate. The medium was replaced after 8 h. Detailed lentiviral infection can be found in the previous methods.^[^ ^46^ ^]^ In brief, 3 µg plasmid, 3 µg assembled plasmid pSPAX2, 2 µg assembled plasmid pMD2G, and 24 µL PEI were mixed into 750 µL opti‐MEM. After 15 min, the mixture was added into 8 mL DMEM medium. The DMEM was replaced after 8 h. Approximately 72 h post‐transfection, the supernatant of cultured HEK293T cells was collected and filtered through a Millex‐GP Filter Unit (Jetbiofil, China) with a 0.45 µm pore size. The lentiviral supernatant was added into PC3, C4‐2 cells. Cells were selected with 2 µg mL^−1^ of puromycin for one week.

### ATP Synthase Enzyme Activity Assessment

Mitochondrial ATP synthase enzyme (Complex V) activity was determined using ATP Synthase Enzyme Activity Microplate Assay Kit (abcam, USA). Briefly, 100 µg protein extraction was fixed onto a specialized 96 well plate. Corresponding to the hydrolyzing ATP to ADP and the oxidation of NADH to NAD^+^ in absorbance at 340 nm was monitored using Tecan SPARK microplate reader.

### Seahorse Mitochondria Stress Analysis

The cell oxygen consumption rate was monitored by Seahorse XF Cell Mito Stress Test Kit and Agilent Technologies XFe24 (Alicelligent, USA). A 100 µL suspension of 1 × 10^5^ mL^−1^ cell was added into 96 well plate. After Seahorse specific test medium rinsing, 500 µL medium was retained. Oligomycin, carbonyl cyanide 4‐(trifluoromethoxy) phenylhydrazone (FCCP), rotenone, and antimycin were added to the detection solution at 30, 60, and 90 min, respectively, to monitor basal respiration, oxidative phosphorylation (ATP‐linked respiration), maximal respiration, and spare respiratory capacity. All compounds were used at a final concentration of 1.0 × 10^−6^ m.

### Transmission Electron Microscopy

PC3, C4‐2 cells were plated on coverslips and treated with 4 × 10^−9^ m docetaxel for 72 h. Cells were immersed in 4 °C 2.5% glutaraldehyde for 2 h, fixed with 1% osmium tetrachloride for 25–30 min, washed twice with PBS. The cells were scraped into EP tubes, and the cell aggregates were dehydrated with successively 50%, 70%, 90%, 100% acetone. After embedding with a freeze sliced embedding agent (Yeasen, China), sections were sliced into many 50 nm pieces using LEICA CM1950 microtome. The pieces were stained with uranyl acetate and lead citrate (Sigma, USA) and viewed at 80kv with a JEOL 1200EX electron microscope, from which digital images were acquired. A magnification of 10000× was used to assess ultrastructure changes in the mitochondria.

### Cell ROS and Mitochondrion ROS Detection

Reactive oxygen species (ROS) were measured following the protocol of the Reactive Oxygen Species Detection Kit (Beyotime, China). Briefly, the probe DCFH‐DA, which is non‐fluorescent and can freely pass through the cell membrane, is hydrolyzed by intracellular esterases to generate DCFH within the cell. Since DCFH cannot penetrate the cell membrane, it remains inside the cell, facilitating probe loading. Intracellular ROS oxidize the non‐fluorescent DCFH to generate fluorescent DCF. Fluorescence intensity was detected using an excitation wavelength of 488 nm and an emission wavelength of 525nm. Similarly, purified mitochondria were used to detect ROS levels following the protocols of the Mitochondria Isolation Kit for Cell and Tissue (Yeasen, China) and the Mitochondrial Reactive Oxygen Species Detection Kit (Beyotime, China).

### Mitochondrial Membrane Potential ∆Ψm Assay

PC3 and C4‐2 cells, stably expressing the vector and *SFT2D2‐TBX19*, were assessed using a mitochondrial membrane potential assay kit with JC‐1 (Beyotime, China) after treatment with 4 × 10^−9^ m docetaxel for 72 h. Cells in a six‐well plate were washed with PBS, followed by the addition of 1 mL of JC‐1 staining solution. The cells were incubated at 37 °C in a cell culture incubator for 20–30 min. After incubation, the JC‐1 supernatant was removed, and the cells were washed twice with JC‐1 buffer. Fluorescence distribution was observed under a fluorescent inverted microscope (Nikon N31373, Japan). When the mitochondrial membrane potential (∆Ψm) is high, JC‐1 aggregates in the mitochondrial matrix produce red fluorescence (excitation wavelength 585 nm). In contrast, at lower mitochondrial membrane potentials, JC‐1 remains in its monomeric form, producing green fluorescence (excitation wavelength 514 nm) when ∆Ψm is low.

### In Vivo Tumorigenesis Assays

For the xenograft tumorigenesis assay, 1×10^7^ PC3 cells (shNC or sh*SFT2D2‐TBX19*) were injected subcutaneously into the right abdomen of 6‐week‐old male BALB/c nude mice using insulin syringes. The PC3 cells were transfected with pLenti‐shNC or pLenti‐shSFT2D2‐TBX19 plasmids to modify their gene expression and then selected with puromycin (2 µg mL^−1^) for 7 d RT‐qPCR was used to verify the knockdown efficiency of the chimeric *SFT2D2‐TBX19*. Once tumors had successfully formed subcutaneously, the volume of the xenografts was measured every 7 d using the formula: volume = length × width^2^ × 0.5. All research protocols were approved by the Ethics Committee of Nanfang Hospital, Southern Medical University (IACUC‐LAC‐20220425‐003).

### Bioinformatics Analysis

For clinical correlation and survival prognosis analysis, mRNA expression profiles and clinical data were collected for prostate cancer patients from the China Population Genome Atlas of Prostate Cancer (CPGEA) database, including 134 PRAD samples and 134 normal samples, as well as 501 PRAD and 52 normal from the Cancer Genome Atlas (TCGA) database. RNA‐seq data in FPKM format were converted to TPM (transcripts per million reads) format for further analysis. Survival analysis was performed using Cox proportional‐hazards model analysis, with the cut‐off value set at the quartile expression level of *TBX19*.

### Statistics Analysis

All data are presented as mean ± SD. Cell numbers for colony formation, migration, and invasion assays were calculated using ImageJ (Fuji) and GraphPad Prism 8.0. The sample size (*n*) for each statistical analysis is provided in the figure legends. Differences in cell proliferation, colony formation, migration, and gene expression between two groups were compared using the Student's t‐test in SPSS 25.0 software. One‐way ANOVA followed by Fisher's LSD was employed to compare differences among more than two groups in SPSS 25.0. IC50 values were normalized to a scale of 0 to 100 using SPSS 25.0 software. Univariate analysis within a general linear model was used to compare regression curves. All statistical tests were two‐sided, with a significance level set at *p* < 0.05.

## Conflict of Interest

The authors declare no conflict of interest.

## Author Contributions

C.H., Z.Z., S.P., and Y.Z. contributed equally to this work. C.H. and Q.W.: investigation, data curation, formal analysis, visualization, writing–original draft, writing–review & editing. Z.Z., S.P., Y.Z., J.J., Z.L.: investigation, formal analysis, writing–review & editing. X.Z., Y.X.: investigation. Y.D., F.L., Q.W., W.T.: conceived the project and critically revised the manuscript, funding acquisition.

## Supporting information



Supporting Information

Supporting Table 1

Supporting Table 2

Supporting Table 3

Supporting Table 4

## Data Availability

Research data are not shared.
