# Supplementary material for: Chimeric SFT2D2‐TBX19 Promotes Prostate Cancer Progression by Encoding TBX19‐202 Protein and Stabilizing Mitochondrial ATP Synthase through ATP5F1A Phosphorylation
Source: Adv Sci (Weinh). 2024 Nov 14;11(48):2408426. doi: 10.1002/advs.202408426 (PMC11672250; doi:10.1002/advs.202408426)
Supplement: Supplementary file 1 — Supporting Information [file ADVS-11-2408426-s005.docx]

**Supplementary:**

**
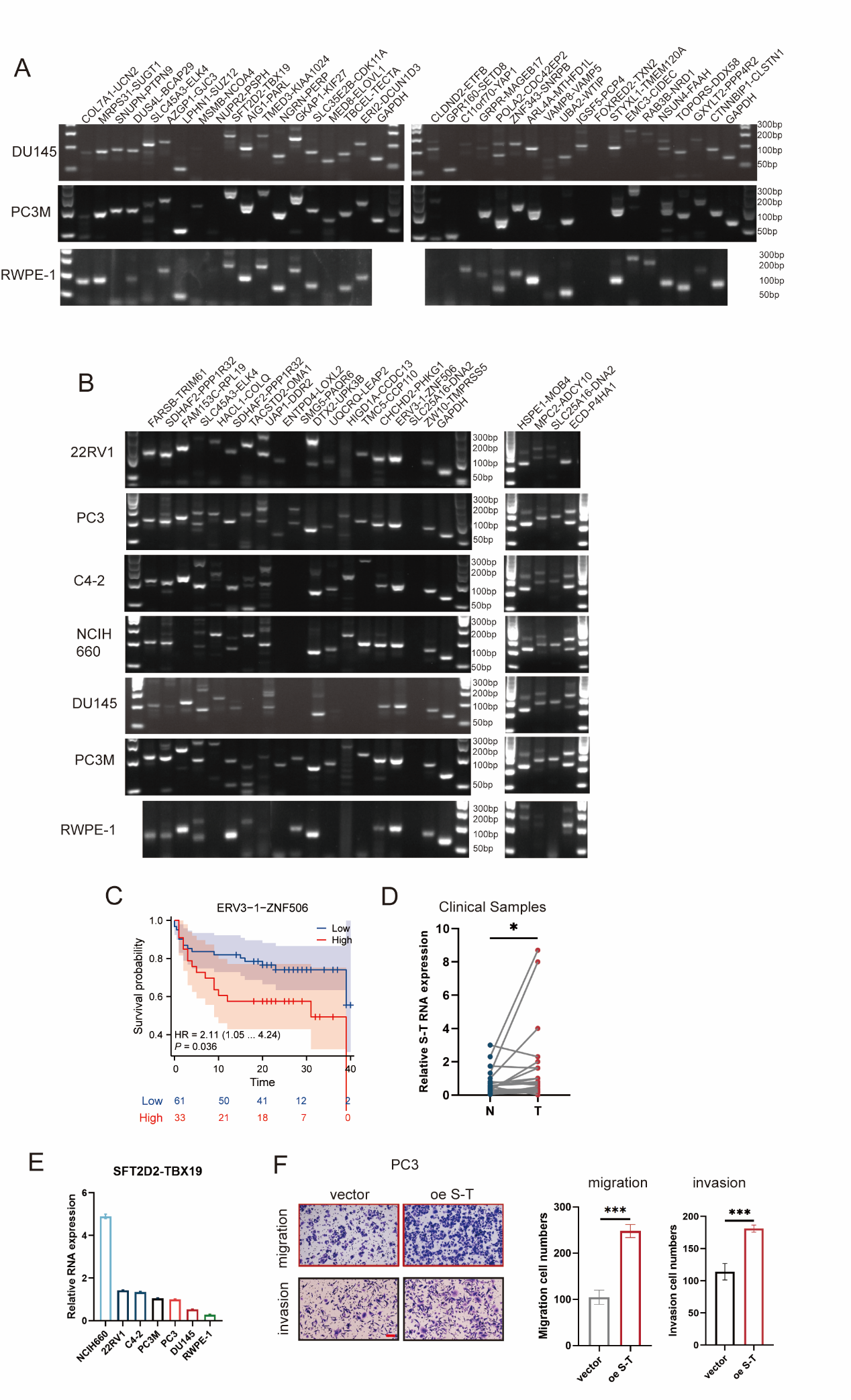
**

Figure S1. (A) Validation of 36 chimeric RNAs in DU145, PC3M, and RWPE-1 cells via agarose gel electrophoresis. (B) Validation of 22 chimeric RNAs in 22RV1, PC3, C4-2, NCI-H660, DU145, PC3M and RWPE-1 cells. (C) Survival probability analysis comparing low and high *ERV3-1-ZNF506* expression groups in the CPGEA database (Cox proportional-hazards model analysis). (D) Transcriptional levels of *SFT2D2-TBX19* between 21 prostate tumors and paired adjacent non-tumor tissues. (E) Relative transcriptional levels of *SFT2D2-TBX19* among NCI-H660, 22RV1, PC3, C4-2, DU145, PC3M, and RWPE-1 cells. (F) Migration and invasion assays of PC3 cells following *SFT2D2-TBX19* overexpression. The scale bar in the lower left corner represents 200μm. Data are represented as mean ± SD. D: n=21, Paired t-test; F, n=3, Student’s t test, *p < 0.05, ***p < 0.001.


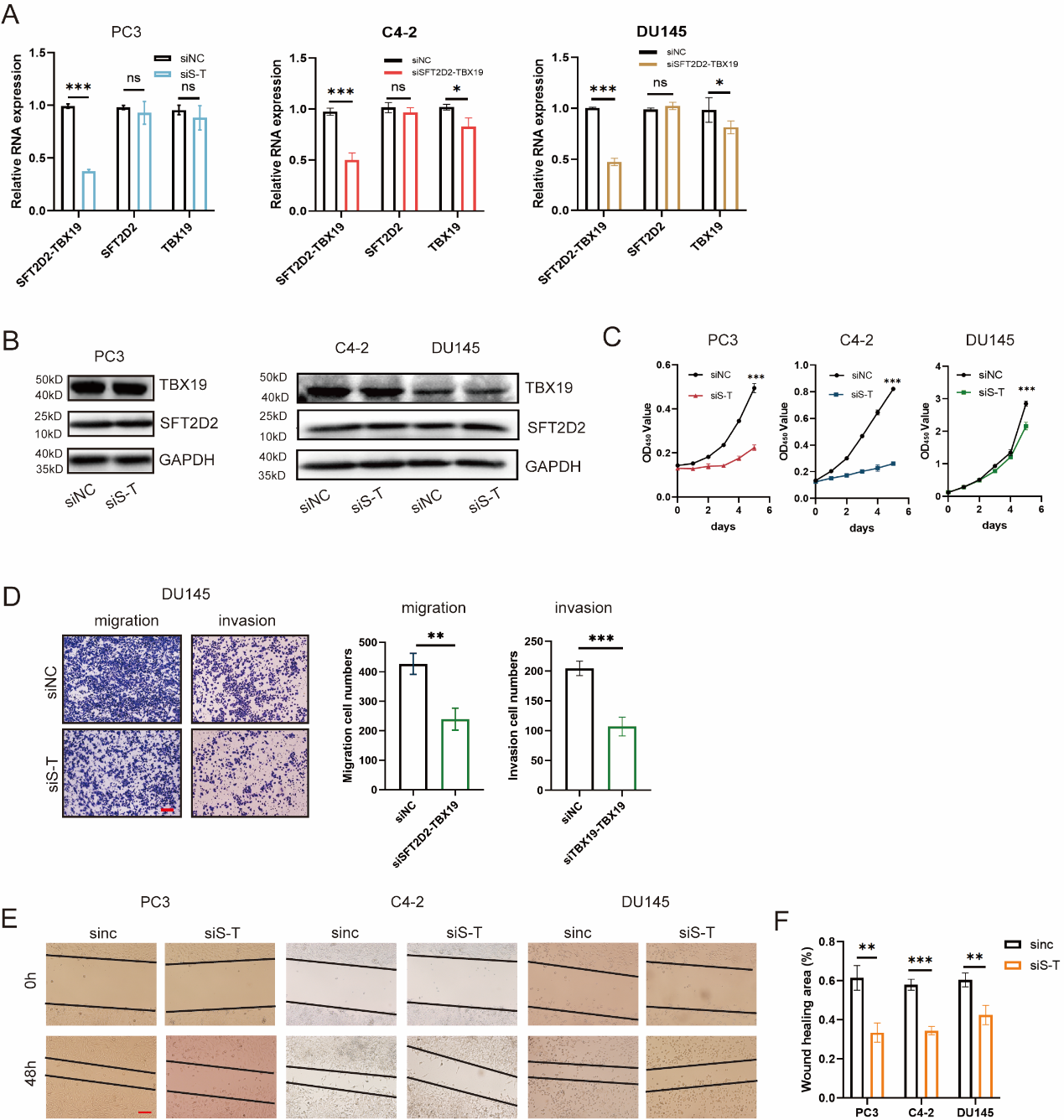


Figure S2. (A) RT-qPCR analysis of chimeric *SFT2D2-TBX19*, parental *SFT2D2*, *TBX19* transcripts in C4-2, PC3 and DU145 cells after *SFT2D2-TBX19* knockdown. (B) Western blot analysis of parental SFT2D2, TBX19 after *SFT2D2-TBX19* knockdown in C4-2, PC3 and DU145 cells. (C) Cell viability testing of PC3, C4-2, DU145 cells after *SFT2D2-TBX19* knockdown. (D) Migration and invasion assays of DU145 cells after *SFT2D2-TBX19* knockdown. The scale bar in the lower left corner represents 200μm. (E)(F) Wound healing and its analysis in PC3, C4-2, DU145 cells after *SFT2D2-TBX19* knockdown. The scale bar in the lower left corner represents 200μm. Data are represented as mean ± SD (n=3). Student’s t test was used to determine statistical significance, ns-no significant differences, *p < 0.05, **p < 0.01, ***p < 0.001.


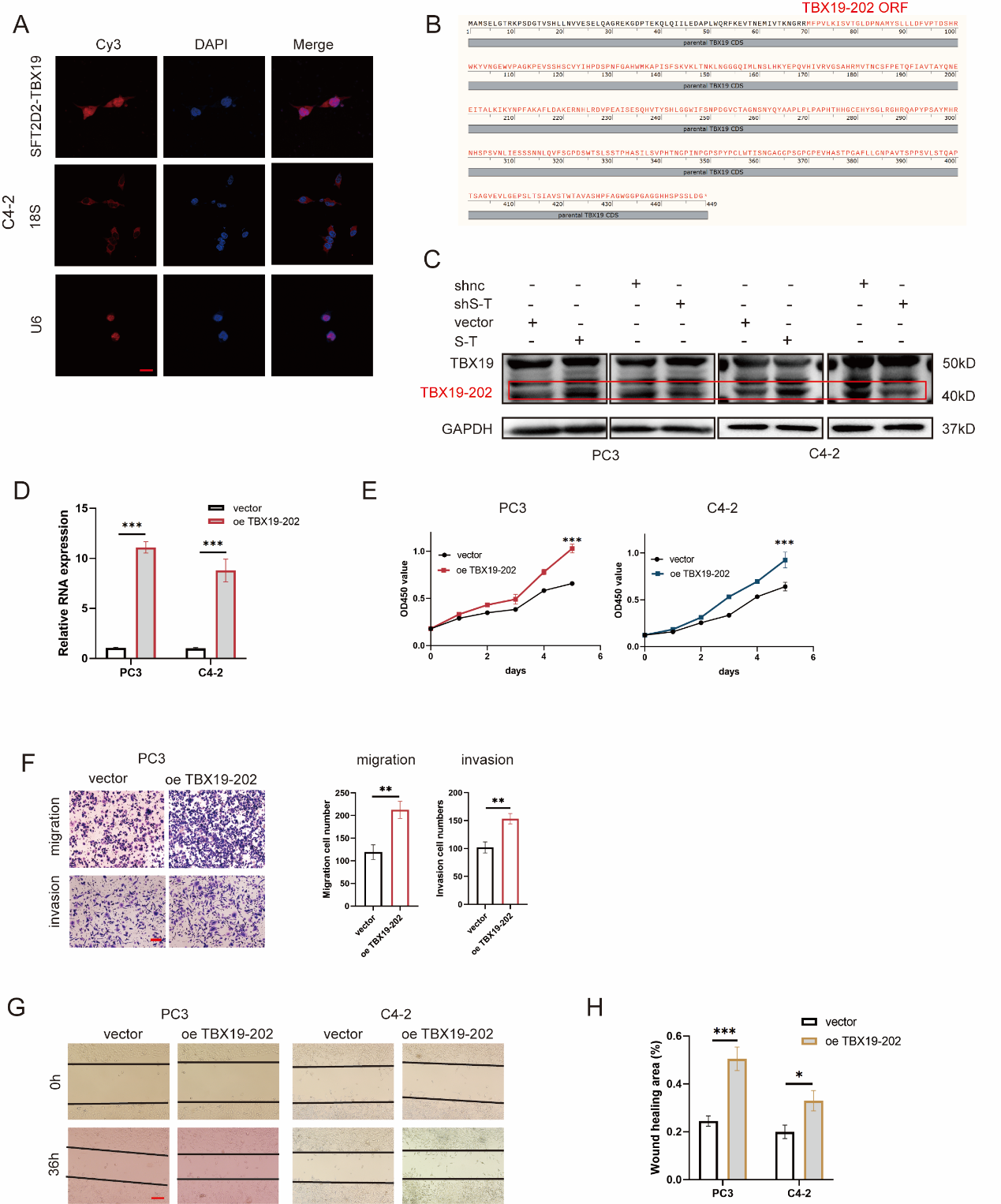


Figure S3. (A) RNA fluorescence in situ hybridization in C4-2 cells for *SFT2D2-TBX19*, with U6 and DAPI as nucleus location references, 18s as a cytoplasm location reference. The scale bar in the lower left corner represents 20μm. (B) ORF comparison of parental TBX19 and TBX19-202. The amino acid sequence is parental TBX19, while the red sequence indicates TBX19-202. (C) Changes in TBX19-202 protein after *SFT2D2-TBX19* knockdown and overexpression in PC3 and C4-2 cells. Red wireframe represents TBX19-202. (D) RT-qPCR validation following TBX19-202 overexpression in C4-2 and PC3 cells. (E) Cell viability testing of PC3, C4-2 cells after TBX19-202 overexpression. (F) Migration and invasion assays of PC3 cells after TBX19-202 overexpression. The scale bar in the lower left corner represents 200μm. (G)(H) Wound healing and its analysis in PC3, C4-2 cells after TBX19-202 overexpression. The scale bar in the lower left corner represents 200μm. Data are represented as mean ± SD (n=3). Student’s t test was used to determine statistical significance, *p < 0.05, **p < 0.01, ***p < 0.001.


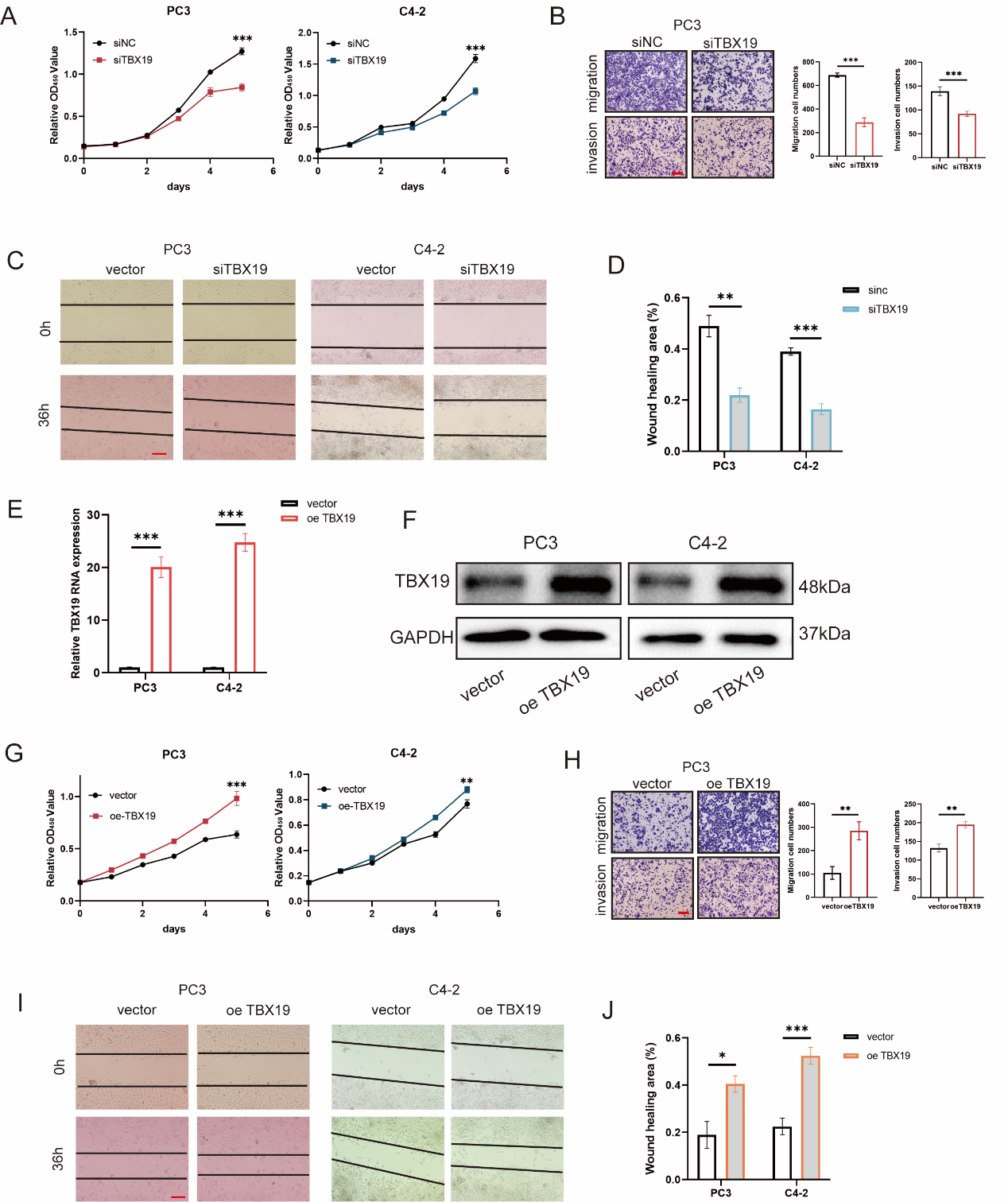


Figure S4. (A) Cell viability testing of PC3, C4-2 cells after parental TBX19 knockdown. (B) Migration and invasion assays of PC3 cells after TBX19 knockdown. The scale bar in the lower left corner represents 200μm. (C)(D) Wound healing and its analysis in PC3, C4-2 cells after TBX19 knockdown. The scale bar in the lower left corner represents 200μm. (E) RT-qPCR analysis of parental TBX19 transcripts in C4-2 and PC3 cells after parental TBX19 overexpression. (F) Western blot analysis of parental TBX19 after TBX19 overexpression in C4-2 and PC3 cells. (G) Cell viability testing of PC3, C4-2 cells after TBX19 overexpression. (H) Migration and invasion assays of PC3 cells after TBX19 overexpression. The scale bar in the lower left corner represents 200μm. (I)(J) Wound healing and its analysis in PC3, C4-2 cells after TBX19 overexpression. The scale bar in the lower left corner represents 200μm. Data are represented as mean ± SD (n = 3 replicates). Student’s t test was used to determine statistical significance, *p < 0.05, **p < 0.01, ***p < 0.001.


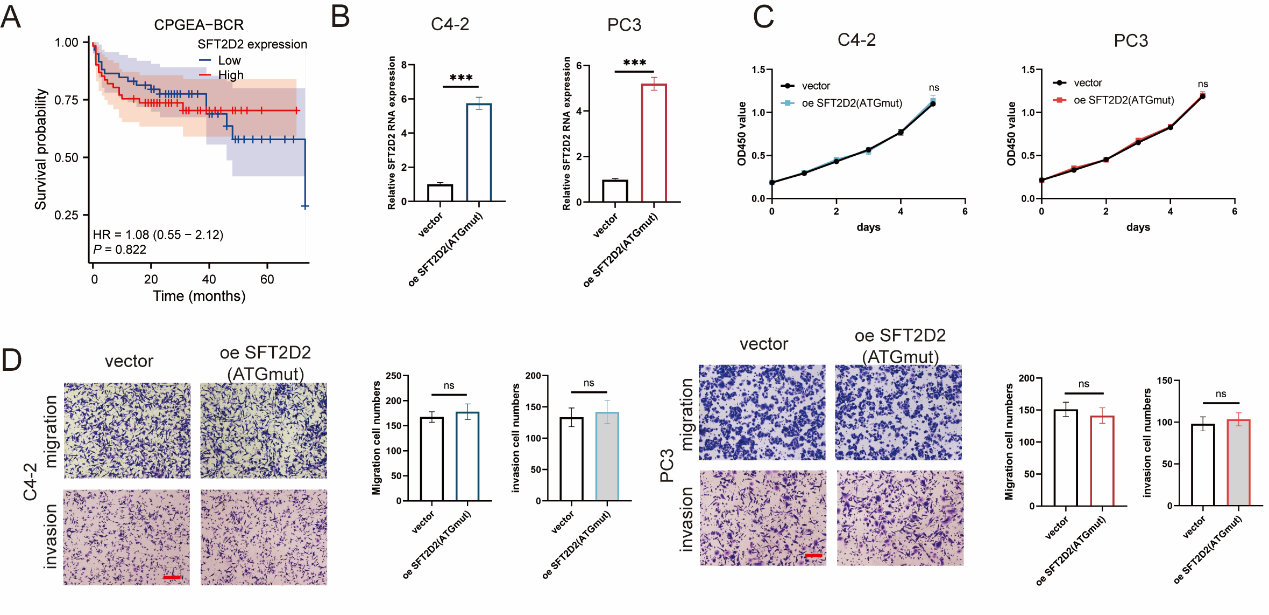


Figure S5. (A) Survival probability analysis comparing low and high *SFT2D2* expression groups in CPGEA database (Cox proportional-hazards model analysis). (B) RT-qPCR analysis of *SFT2D2* (ATGmut) transcripts in C4-2, PC3 cells after *SFT2D2* (ATGmut) transfection. (C) Cell viability testing of PC3, C4-2 cells after *SFT2D2* (ATGmut) overexpression. (D) Migration and invasion analysis of C4-2, PC3 cells after *SFT2D2* (ATGmut) overexpression. The scale bar in the lower left corner represents 200μm. Data are represented as mean ± SD (n = 3 replicates). Student’s t test was used to determine statistical significance, ns-no significant difference, ***p < 0.001.


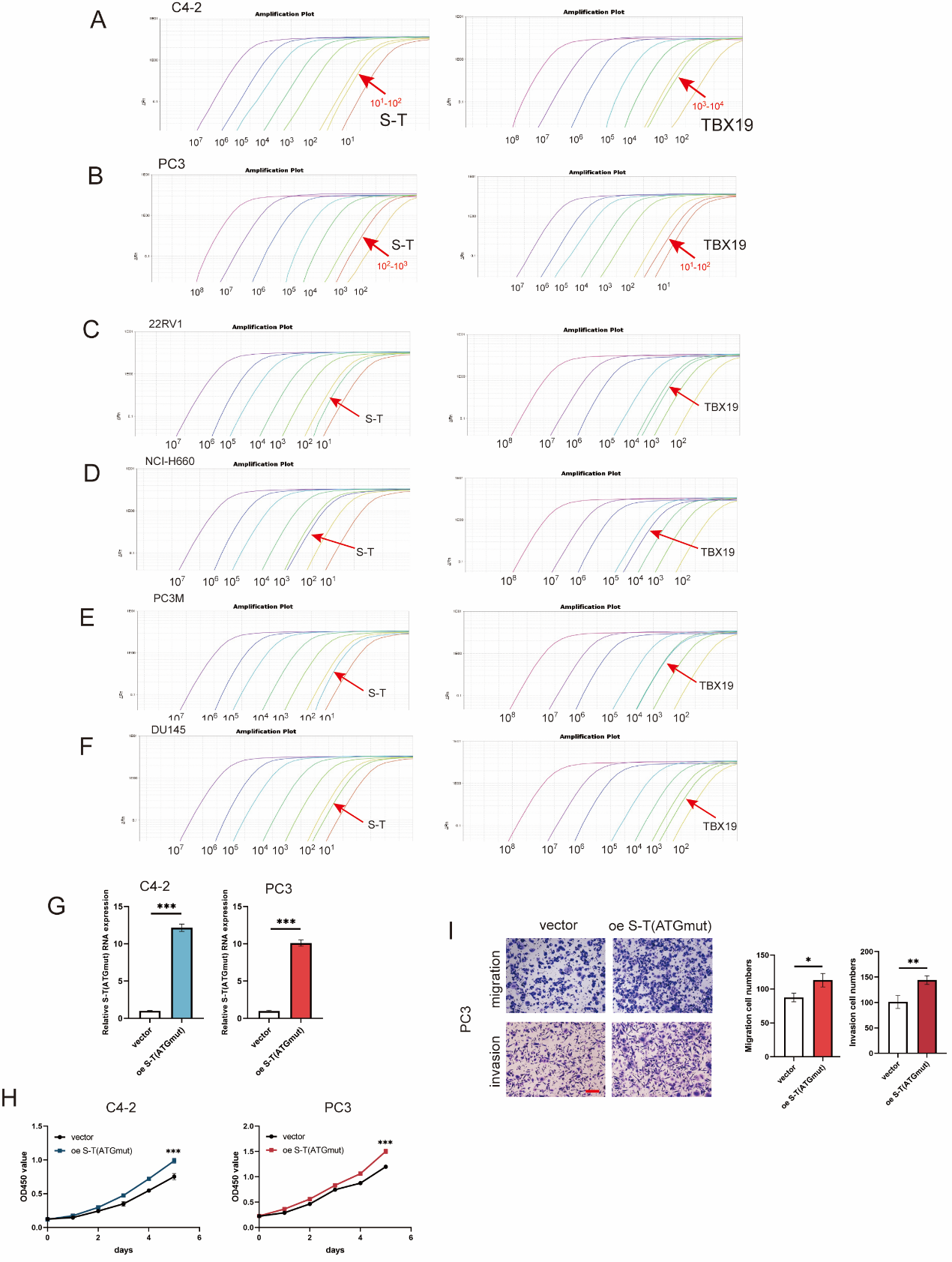


Figure S6. (A-F) The relative transcriptional levels of chimeric *SFT2D2-TBX19* and parental *TBX19* in C4-2, PC3, 22RV1, NCI-H660, PC3M, and DU145 cells. Red arrows point to respective amplification curves. Standard curves were created using precisely calculated plasmid concentration in the PCR system; for example, the molecular weight of plasmid pCDH-*SFT2D2-TBX19* is about 660 × 11754bp = 7,757,640Da, 1pg of this plasmid is 10^-12^ × 6.02 × 10^23^ / 7,757,640 = 77,600 copies. (G) RT-qPCR analysis of *SFT2D2-TBX19* (ATGmut) transcripts in C4-2, PC3 cells after *SFT2D2-TBX19* (ATGmut) transfection. (H) Cell viability testing of PC3, C4-2 cells after *SFT2D2-TBX19* (ATGmut) overexpression. (I) Migration and invasion analysis of PC3 cells after *SFT2D2-TBX19* (ATGmut) overexpression. The scale bar in the lower left corner represents 200μm. Data are represented as mean ± SD (n = 3 replicates). Student’s t test was used to determine statistical significance, *p < 0.05, **p < 0.01, ***p < 0.001.


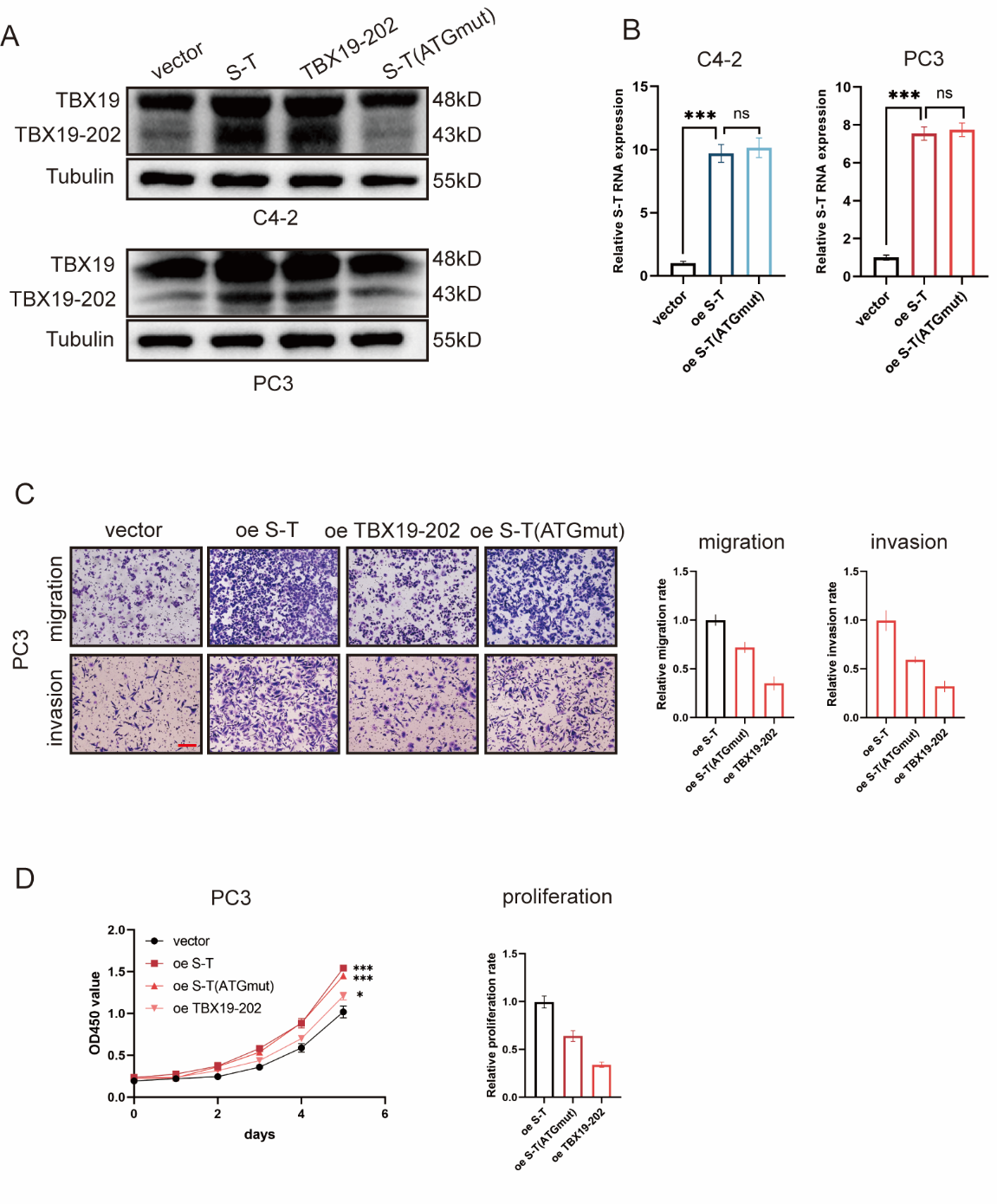


Figure S7. (A) Detection of TBX19 and TBX19-202 protein after transfection with vector, wild-type *SFT2D2-TBX19*, TBX19-202, and *SFT2D2-TBX19* (ATGmut) in C4-2, PC3 cells. (B) The relative transcriptional levels of chimeric *SFT2D2-TBX19* or *SFT2D2-TBX19* (ATGmut) in C4-2, PC3 cells after transfection with vector, wild-type *SFT2D2-TBX19*, and *SFT2D2-TBX19* (ATGmut). (C) Migration and invasion analysis of PC3 cells after overexpression of vector, wild-type *SFT2D2-TBX19*, TBX19-202, and *SFT2D2-TBX19* (ATGmut). The scale bar in the lower left corner represents 200μm. (D) Cell viability testing and proliferation rate analysis of PC3 cells transfected with vector, wild type *SFT2D2-TBX19*, ATG mutated *SFT2D2-TBX19*, and TBX19-202 separately. Data are represented as mean ± SD. B, D: n = 3, one‐way ANOVA with Fisher's LSD. ns- no significant difference, *p < 0.05, **p < 0.01, ***p < 0.001.


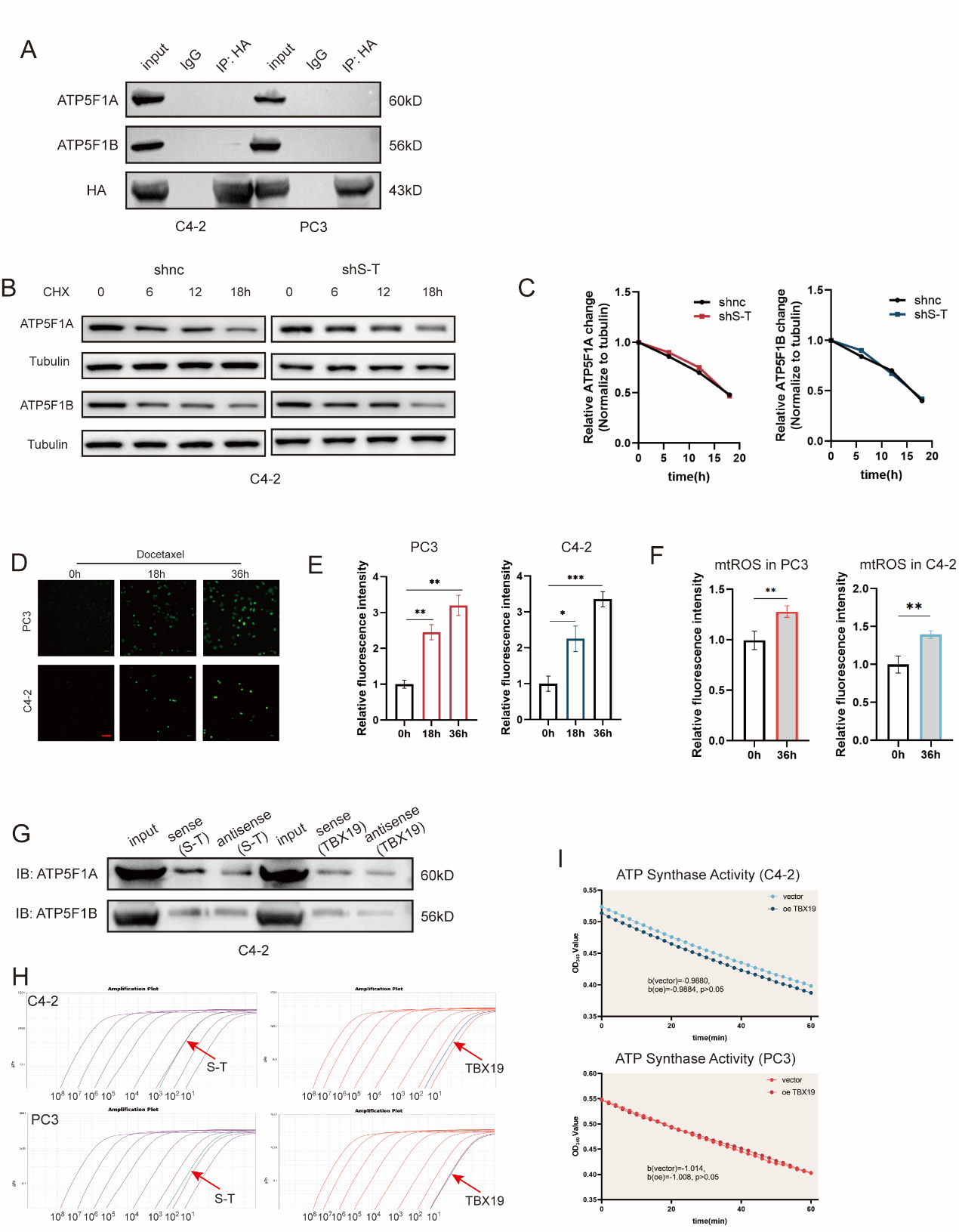


Figure S8. (A) Co-immunoprecipitation analysis between TBX19-202-HA and ATP5F1A/B. (B, C) Degradation of ATP5F1A and ATP5F1B proteins and their analysis after 50 μg/mL cycloheximide treatment in shNC and sh*SFT2D2-TBX19* C4-2 cells. Beta-tubulin works as an internal reference protein. (D, E) Assessment of cellular ROS intensity via fluorescence with 488nm excitation and 525nm emission wavelength. (F) Mitochondrial ROS detection in purified mitochondria from PC3 and C4-2 cells after docetaxel treatment for 0 and 36h. (G) Western blot analysis using anti-ATP5F1A, anti-ATP5F1B for pulldown proteins under the condition of same mol quantity of probes *SFT2D2-TBX19* sense, antisense and TBX19 sense, antisense in PC3 cells. (H) The relative levels of chimeric *SFT2D2-TBX19* and parental TBX19 transcripts in mitochondria from C4-2 and PC3 cells. Red arrows point to respective amplification curves for *SFT2D2-TBX19* and parental *TBX19*. (I) Analysis of ATP synthase enzymatic activity in PC3 and C4-2 cells after *TBX19* overexpression. Data are represented as mean ± SD. E: n = 3, one‐way ANOVA with Fisher's LSD. F: n = 3, Student’s t test, **p < 0.01, ***p < 0.001.


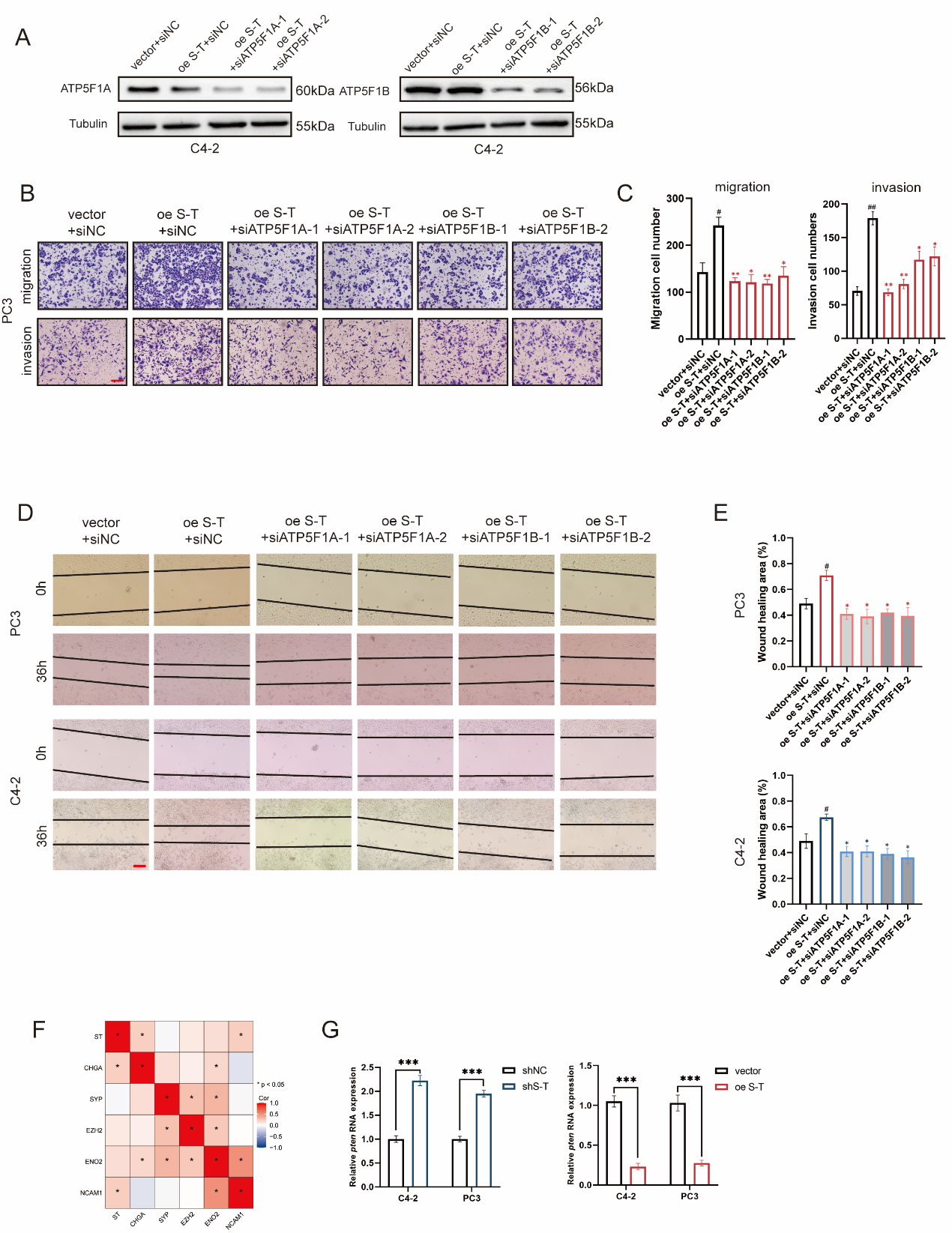


Figure S9. (A) Validation of ATP5F1A and ATP5F1B knockdown at the protein level in C4-2 cells. (B-C) Migration and invasion assays of PC3 cells with *SFT2D2-TBX19* overexpression after ATP5F1A or ATP5F1B knockdown. The scale bar in the lower left corner represents 200μm. (D-E) Wound healing and its analysis in PC3, C4-2 cells with *SFT2D2-TBX19* overexpression after ATP5F1A or ATP5F1B knockdown. The scale bar in the lower left corner represents 200μm. (F) Correlation heat map among *SFT2D2-TBX19*, CHGA, SYP, EZH2, ENO2, and NCAM1 in CPGEA database. (G) RT-qPCR analysis of *pten* transcripts in C4-2 and PC3 cells after *SFT2D2-TBX19* knockdown and overexpression. Data are represented as mean ± SD. C, E: n = 3, one‐way ANOVA with Fisher's LSD. ns- no significant difference, *p < 0.05, **p < 0.01-compared with oe *SFT2D2-TBX19*+siNC; ^#^p<0.05, ^##^p<0.01-compared with vector+siNC; G: n=3, Student’s t test, **p < 0.01, ***p < 0.001.
